# Spontaneous innovation of hook-bending and unbending in orangutans (*Pongo abelii*)

**DOI:** 10.1038/s41598-018-34607-0

**Published:** 2018-11-08

**Authors:** I. B. Laumer, J. Call, T. Bugnyar, A. M. I. Auersperg

**Affiliations:** 1 Department of Cognitive Biology, University of Vienna, Althanstr. 14, 1090 Vienna, Austria; 2 School of Psychology & Neuroscience, University of St. Andrews, Westburn Lane, St. Adrews, UK; 3 Messerli Research Institute, University of Veterinary Medicine (other partner institutions: University of Vienna, Medical University of Vienna), Veterinärplatz 1, 1210 Vienna, Austria

## Abstract

Betty the crow astonished the scientific world as she spontaneously crafted hook-tools from straight wire in order to lift a basket out of vertical tubes. Recently it was suggested that this species’ solution was strongly influenced by predispositions from behavioural routines from habitual hook-tool manufacture. Nevertheless, the task became a paradigm to investigate tool innovation. Considering that young humans had surprising difficulties with the task, it was yet unclear whether the innovation of a hooked tool would be feasible to primates that lacked habitual hook making. We thus tested five captive orangutans in a hook bending and unbending task. Orangutans are habitually tool-using primates that have been reported to use but not craft hooked tools for locomotion in the wild. Two orangutans spontaneously innovated hook tools and four unbent the wire from their first trial on. Pre-experience with ready-made hooks had some effect but did not lead to continuous success. Further subjects improved the hook-design feature when the task required the subjects to bent the hook at a steeper angle. Our results indicate that the ability to represent and manufacture tools according to a current need does not require stereotyped behavioural routines, but can indeed arise innovatively. Furthermore, the present study shows that the capacity for hook tool innovation is not limited to large brained birds within non-human animals.

## Introduction

Tool-manufacture can be found in distantly related species but its occurrence is extremely rare^1^. Innovative tool manufacture differs from stereotyped tool manufacture in that incorporates the active production of an old tool from a novel material, the modification of an old tool-type for a novel purpose or the invention of a completely new tool-type and is thus not part of the animal’s habitual repertoire^2,3^. Although innovative tool manufacture is often associated with advanced cognitive processing, the underlying mechanisms, e.g. transferring motor pattern vs. inhibiting old behavior, may be different^4^. New forms of innovative tool manufacture are likely to represent the most sophisticated types of technical innovations expressed by animals. The hook bending/unbending paradigm is well suited to investigate innovative tool manufacture: To be successful an animal has to simultaneously execute several unrewarded steps with precise motor-control thereby keeping the final goal in mind^5,6^.

Hooks can be broadly defined as tools with a curved end in which the curvature constitutes a necessary functional feature that allows the subject to lift the basket out of the tube. In human evolution, they appear relatively late in the prehistoric record. There is definite evidence for the manufacture of a fish hook from East Timur dating back ca 16,000–23,000 years^7^, although findings in central Africa suggest that harpoon-like objects could date back as early as ca. 60,000 years^8^. Nevertheless, some apes, notably some populations of chimpanzees, have been observed to break of twigs with side branches to catch and retrieve other branches that are otherwise out-of-reach (e.g.^1^). While it is unclear whether these apes employed the side branches of twigs as hooks in a goal directed manner it is possible that branch-hauling tools represents one of the earliest and simplest hooks made and used by great apes, including some of our ancestors.

The ability to spontaneously and actively bend a hooked tool from a pliant material in order to lift a baited basket with a handle out of a vertical tube, has prominently been studied in two corvids, one parrot and human infants. In 2002 the New Caledonian crow Betty was accidently observed to spontaneously craft a hook tool out of a straight piece of wire, after her mate had flown off with the provided ready-made hook-tool^9^. Although Betty’s behaviour was for a long time considered a prime example of innovative tool manufacture in animals, a recent study showed that wild-caught New Caledonian crows employ the same motor action sequence that Betty used during hook bending to adjust the shaft of their tools during habitual tool making, thereby calling the innovative strength required to achieve her original solution to the original hook bending task into question^10,11^. Nevertheless, note that Betty’s behaviour still qualifies as innovative tool manufacture within our original definition since she applied an existing object modification to solve a novel problem and used a novel material while doing so. Another corvid, the rook and a parrot, the Goffin’s cockatoo were similarly capable of finding a solution to the hook-bending task^12,13^. In contrast to NCCs, neither of the previous species are specialized tool users, therefore their behaviour cannot be as easily influenced by inherited routines from habitual tool manufacture. Goffin’s cockatoos were even able to invent hooked and straight tools by bending and/or unbending wire, while lacking any pre-experience with ready-made hooks (in contrast to the rooks and the New Caledonian crows, which had previously received experience in using a ready-made hook tool in the same context^9,12,13^). Therefore, the innovative strength required for solving the hook bending task is expected to be lower if a subject has many inherited predispositions for solving the task and/or more experience with the task affordances.

Interestingly the hook bending paradigm represents one of the rare cases in which comparative cognition was turned upside down^14^: a test that was originally developed from an accidental observation of a bird, that was later tested on other avian species was finally also employed to test the development of tool innovation in human infants^15–19^. Surprisingly, when confronted with a straight piece of wire, only around 5% of the children under five years of age and only about 50% of the eight-year-old children were able to solve the problem. This was unexpected since children seemed to possess all relevant knowledge about the properties of the materials used and the physics of the task to succeed: to ensure that subjects knew that the wire was pliable and retains its form after bending, they received manipulation exercises prior to the experiment^15^ (Experiment 3;^16^, Experiment 1). Furthermore, the children seemed to know that the hooked tool was functional, since when given a choice between a straight and a hooked wire, most children as young as four years chose the functional tool^15^ (Experiment 1). When given the pre-experience of using a premade hook-tool the majority of four to five-year-old children were able to solve the hook-bending problem^17^. Additionally, when given demonstrations on how to bend a hook tool after an initial failure in the original hook-bending task, children were able to solve the original problem, although they were not given demonstrations on how to use it^15,16^. Tool innovation was therefore identified as limiting step, since almost all children solved the task after receiving a hook-bending demonstration. Cutting and colleagues^16,18^ suggested that hook-bending may represent an ill-structured problem: the means of getting from the beginning to the end of the task cannot be determined from the structure of the problem itself and the actors have to thus structure the executive process on their own. The fact that we humans take this long to develop the capacity for this type of tool innovation, coupled with us being the only habitually hook-tool using and hook-manufacturing primates^1^, suggests that the ability to innovate hooks from pliant material may very well be limited to humans within primates.

Nevertheless, goal-oriented modification of pliable materials has been studied prior to the existence of the avian hook bending paradigm: Povinelli *et al*.^20^ tested whether seven juvenile chimpanzees would straighten one end of an S- or C-shaped wire in order to push it into a hole to obtain a reward. One chimpanzee was able to successfully manipulate the wire, but only after receiving demonstrations by the experimenter and additionally being trained to unbend the wire in absence of the apparatus. Furthermore Klüver (1937) and Anderson & Hennemann (1994) tested whether capuchin monkeys (n = 1^21^; n = 2^22^) could unbend a circular wire in order to insert it into a honey-dipping apparatus. One male straightened and used the wire as a tool. But as Weir & Kacelnik^23^ pointed out, the authors did not provide enough information to draw conclusions whether the capuchin unbend the wire in an intentional goal-directed way.

Orangutans are habitual tool users that show a diverse repertoire of using and making tools in the wild as well as in captivity (e.g.^1,24–27^). Despite two anecdotal field observation of orangutans using a raking-tool for locomotion (both cases of branch hauling as described above for chimpanzees), there is no evidence of habitual hook-tool use nor manufacture in wild or captive orangutans: in order to reach a nearby tree branch after failed attempts to reach it manually, a wild male Sumatran orangutan was observed breaking off a live branch of one meter length and seemingly trying to rake the respective branch^28^. After two failed attempts, he dropped it and broke off a longer branch of approximately 2.5 meters in length and used it successfully to rake the target branch while tree swaying, drew it closer and transferred to the adjacent tree. The branch used was described as relatively straight with several leafy branches bent towards the apex and was not reshaped after detaching it. A similar use of a branch for transferring from one tree to the other was once observed in an unhabituated female with infant in Tuanan, Borneo^29^. It is assumed that this behaviour might have been triggered by the presence of the observer, leading the ape to use an alternative route to avoid getting closer to the human observer. Since there are no reports of any other branch-hook tool use in the wild (e.g.^1^; note that in this example the apex of the tool was not actively shaped by the animals: the orangutans did not actively modify the tool after breaking it off the tree. Its natural shape with the leafy branches at the working end was already functional for the purpose of raking close the branch), this behaviour was considered to be innovative^29^. Experiments in the lab suggest that orangutans are able to encode and mentally compare tool length and the tool’s relation to the distance of the desired object^30^. There is also evidence that orangutans improve a tool’s design through active modification: observations on wild orangutans showed that probing tools that were manufactured in the foraging context (insect extraction and honey dipping) were modified by stripping the bark with the teeth and by chewing or splitting the tip. From 201 tools only one tool was forked and most tools were straight and unbranched^31^. Chimpanzees observed in the Goualougo triangle of Congo show similar tool modifications. Since this brush tipped tools have been experimentally shown to be more effective than unmodified sticks, the authors suggest that the split end is a deliberate design feature^32^. Further two out of three orangutans of a captive group manipulated probing tools by stripping of the leaves of branches but leaving one to several leaves on the tip, in order to probe in an artificial termite mound^27^. It is conceivable that this might increase the functionality of the tool, as more food could be obtained by increasing the tool’s surface.

So far humans are the only primate species that have been tested on the hook-bending task. By testing habitually tool-using orangutans, we aim to contribute to fill the gap in the existing comparative framework on this matter. Note that the hook innovation task is harder than other tasks in which subjects were given a ready-made hook or a rake to obtain an out-of-reach food item^9,12^. It is therefore not a foregone conclusion that orangutans would be able to solve the hook innovation task. Furthermore, we aim to answer the question of whether the capacity for hook tool innovation is limited to large-brained birds, which have previously shown sophisticated problem solving skills rivalling and/or even outperforming the great apes in some tasks. More specifically, we addressed the following goals: (i) To investigate whether task-naive orangutans could innovate a hook out of a straight wire when confronted with the hook-bending problem, as previously tested on corvids, cockatoos and human infants. In order to maintain some comparability between studies/species we apply the same methodology as has recently been used on Goffin cockatoos, a non-habitually tool using parrot^13^. (ii) To target the context independent flexibility underlying tool-bending in our subject species, we additionally tested all subjects in an unbending task, in which the reward was inside a horizontal tube and a bent wire, too short to reach the reward in this form, was provided. (iii) We further aim to explore the effect of pre-experience with hook tools in a scaffolding manner. From all species tested so far, the effect of pre-experience has only been investigated in the Goffin cockatoos^13^. In the latter it had some effect, but was not essential for task success. (iiii) To investigate whether successful subjects are able to improve the hook-design feature, we run a follow up test in which the basket is attached to the bottom of the tube with Velcro fastener. This modification requires the subjects to bent the wire at a steeper angle to retrieve the basket, otherwise the handle of the basket would slip off the hooked end of the tool. Orangutans are a promising candidate species, as there already exists two observations of hook-tool use in the context of locomotion in the wild^28,29^. Based on high innovation rates of great apes in the wild^33^ as well as in the lab (e.g. floating peanut experiment^34^, we expect that orangutans could well be able to solve both problems. We further expect them to be able to improve the tool-design feature, since orangutans are known to modify stick tools in the wild^31^.

## Methods

### Subjects, housing and experimental histories

Four adult females (Pini, Raja, Dokana, Padana), one adult male (Bimbo) housed at the Wolfgang Köhler Primate Research Centre in the Leipzig Zoo participated in this experiment. An additional 6-year-old female orangutan (Tanah) was not initially included in the study, but participated in one of the tasks after her mother (Dokana) stopped participating in it. The orangutans were maintained in an enriched indoor- and outdoor enclosure (for more details on the housing conditions see SI, chapter a). The research was approved by the ethics joint committee of the Max Planck Institute for Evolutionary Anthropology and Zoo Leipzig and the methods were carried out in accordance with the relevant guidelines and regulations. Subjects were tested individually, participated on a voluntary basis and were never food or water deprived. All animals had experience with a variety of physical cognitive tasks. All subjects except Raja and Tanah had experience in raking an out-of-reach reward on a horizontal platform by using a raking tool (the material and its physical appearance differed strongly: the head of the rake was made out of a wood board (30 × 1 × 1 cm) that was attached to a wooden rod^35^; (for detailed information on subjects and experimental histories see SI, chapter a). Two subjects (Pini and Dokana) had experience in pulling close a bottle of grape juice hanging from the ceiling by the means of a rigid unbendable metal hook (35 cm in length^36^). None of the subjects had used a raking tool in any other context than mentioned above nor had seen or used a hook tool made of a pliant material before.

### Apparatus

**Figure 1:**
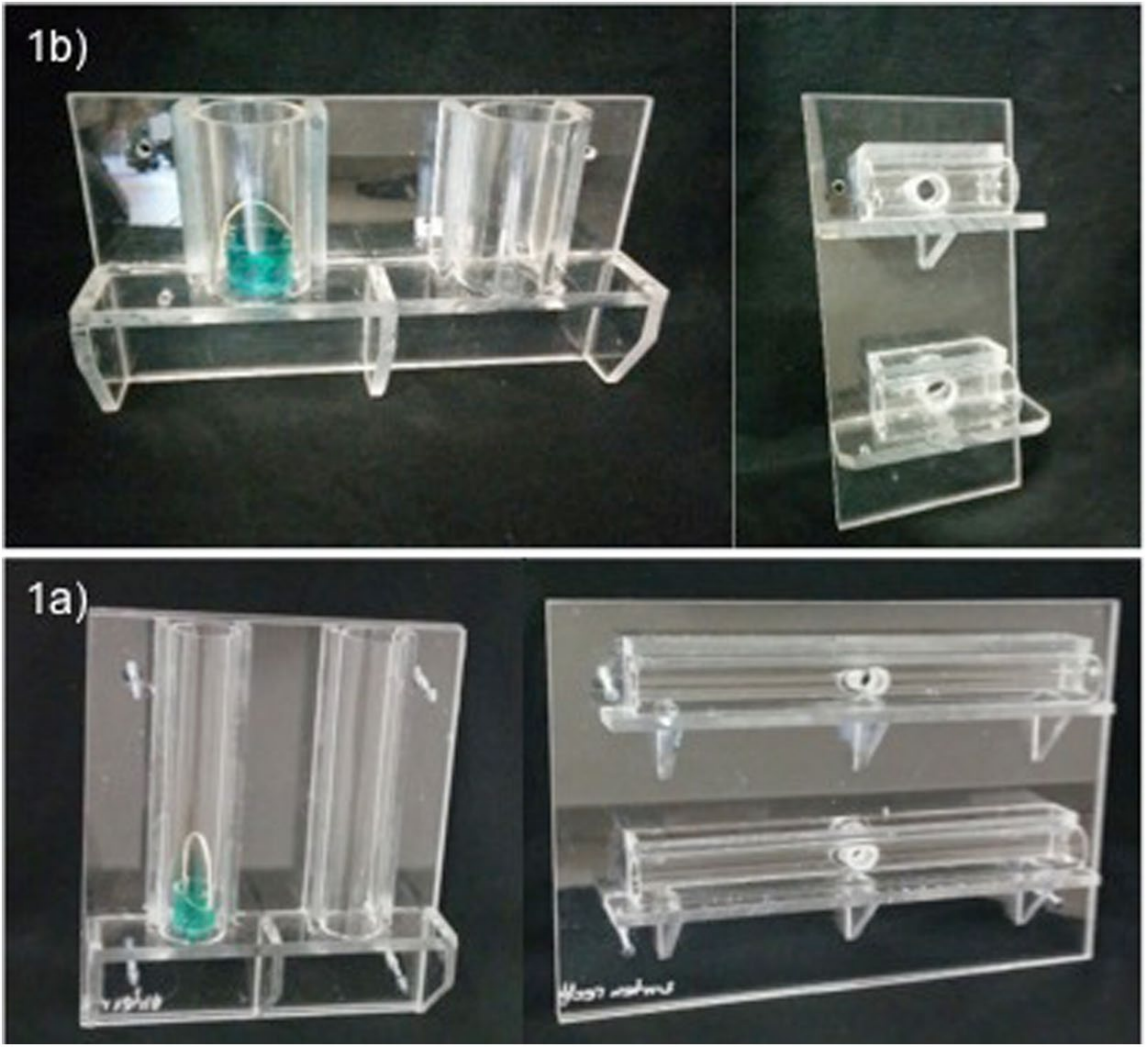
(**b**) Left: Picture of the habituation apparatus for the hook-bending condition with inserted basket; Right: Picture of the habituation apparatus for the unbending condition (**a**). Left: Picture of the hook-bending apparatus with inserted basket; Right: Picture of the unbending apparatus.

We used two apparatuses corresponding to the two tasks. The bending task apparatus consisted of two vertically oriented 22-cm tubes mounted on a Plexiglas panel that was in turn fastened to the cage mesh (see Fig. 1a, left). A banana flavored pellet was placed inside a basket inside one of the tubes, the other tube remained empty and served as a no-food control. The basket could be extracted by catching its loop (with a hook) and pulling it vertically in the direction of the tube’s opening. Subjects had at their disposal two novel materials: a woolen string and an aluminum wire both measuring 29 cm in length. Only the wire was suitable to extract the basket provided one of its ends was fashioned into a hook. The woolen string served as a distractor material. The unbending task apparatus consisted of two horizontally oriented 40-cm tubes mounted on a Plexiglas panel that was also fastened to the cage mesh. A banana flavored pellet was placed inside one of the tubes (equidistant from both tube openings) while the other tube remained empty. The string and the wire were only 26 cm in length to ensure that the v-bent wire was too short to reach the reward located in the middle of the tube (see Fig. 1a, right). Prior to each test, subjects faced a habituation apparatus identical to the test apparatus except that their tubes were shorter (10 cm) so that the reward could easily be extracted with their fingers by lifting the basked (bending task; see Fig. 1b, left) or poking the banana pellet out (unbending condition; see Fig. 1b, right). This experience was considered necessary because neither the Goffin cockatoos^13^ nor the orangutans in present study had seen a basket before that could be pulled up by the handle. Note that both corvid species^9,12^ had prior experience with a ready-made tool and the same testing apparatus before the actual test started.

#### Design

All subjects received both tasks except Dokana and Tanah, who only completed one task. Padana and Pini (and Tanah) started with the bending task whereas Dokana, Raja and Bimbo began with the unbending task. We divided the subjects into two groups depending on their experience using the metal unbendable hook prior to the start of the experiment (for details see above). Non-experienced orangutans (Padana, Raja, Bimbo; Group N) received stepwise scaffolding when they failed to solve the original version of the tasks (see below) whereas the experienced group (Pini & Dokana; Group E) received no such scaffolding but received the same number of sessions as non-experienced subjects (see SI for details about how many trials each individual received). Raja, Bimbo (both non-experienced) and Dokana (experienced) received the unbending task followed by the bending task whereas Padana (non-experienced) and Pini (experienced) received the reverse order.

#### Procedure

Prior to the test, subjects received two habituation sessions of 20 trials (with each apparatus) in which they could extract food from the habituation apparatuses using their fingers. The position of the reward was semi-randomized across sessions. Furthermore, subjects observed the experimenter warp the wire and the string around a pen a total of nine times in random order on two consecutive days. Afterwards subjects received two 15-min sessions in which they could freely manipulate the wire and the string.

Upon completing the habituation sessions, subjects received the bending and unbending task. Subjects received up to three versions of each task depending on their proficiency level. Subjects began with the hardest version of the task and if unsuccessful they subsequently received the easier versions. We used the following three versions of the task:

#### Unmodified materials (U)

In the bending task we placed the straight string and the straight wire on the right and left side of the apparatus whereas in the unbending task we placed a bent wire and a bent string (both bent at a 45° angle) on the right and left side of the apparatus.

#### Pre-prepared materials (PP)

In the bending task subjects received a ready-made hook tool and a similar shaped string placed next to the apparatus. The orientation of the bent end was randomized across trials. In the unbending task a straight wire and string were placed alongside the apparatus.

#### Pre-inserted and pre-prepared materials (PI&PP)

In the bending task a ready-made hook tool and string were already pre-inserted with the hook caught in the basket’s handle. In the unbending task a straight wire and string were pre-inserted into the tube.

**Figure 2:**
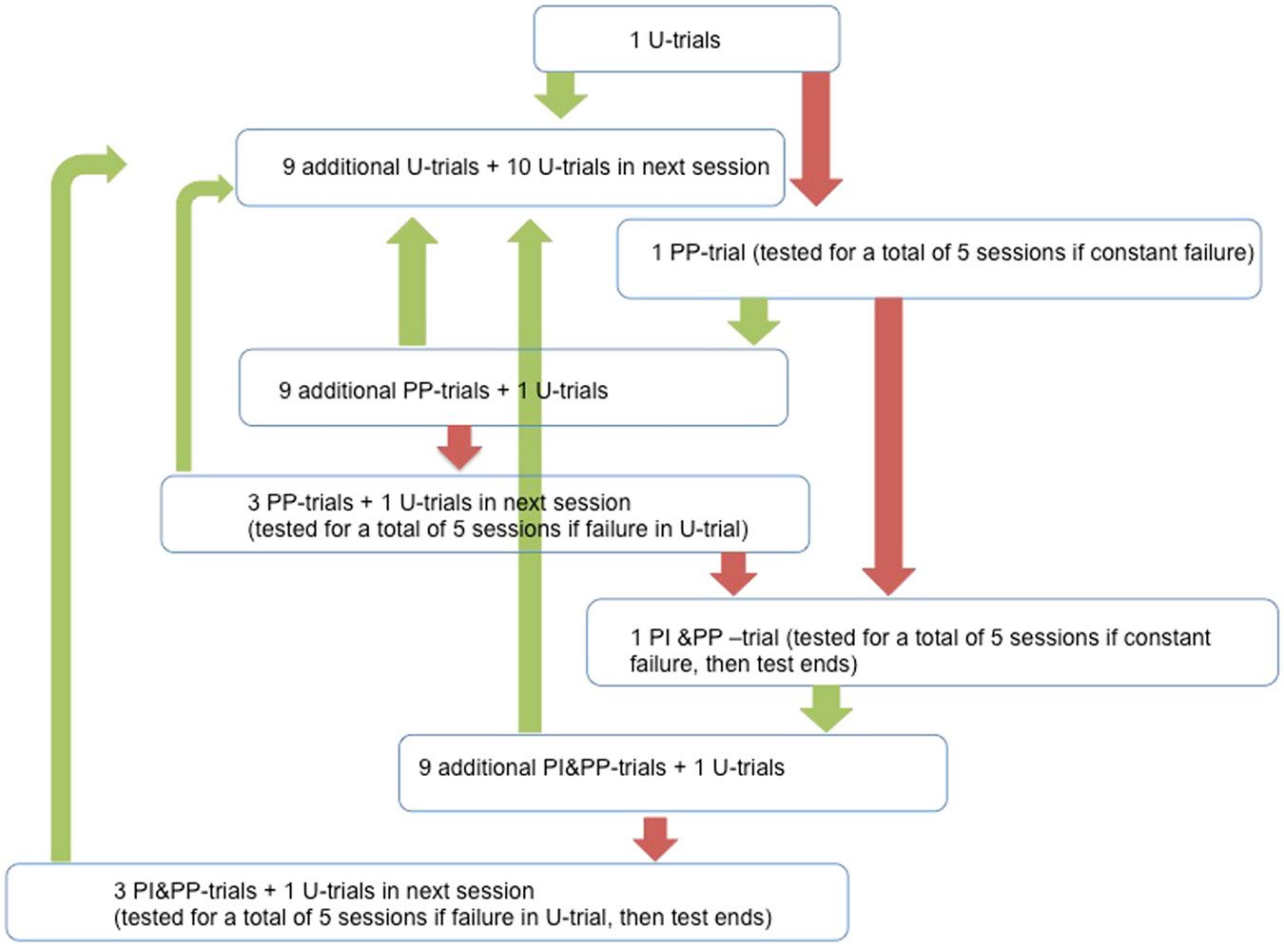
Flowchart of the procedure. Green arrows indicate success (subject operates the apparatus by using the tool), red arrows indicate failure (subject fails to retrieve the reward). Detailed description see directly below in the text.

All subjects began with the U version of each task. If successful on the first trial, they received up to nine additional trials (see Fig. 2). Upon completing 10 trials in a row during the first session, they received additional trials until they completed 20 trials within two out of three consecutive sessions. If they failed the U trial, i.e., they were unable to retrieve the reward within 15 minutes, subjects of the non-experienced group received the PP version in the next test session. If they succeeded 10 times within one session, they received one U trial. If they failed this trial, subjects received three PP trials (which they had passed earlier) followed by one U trial in the next test session. If successful in the U trial, subjects received up to nine additional U trials depending on task success. Testing continued until subjects continuously succeeded in 20 trials within two out of three consecutive sessions. If they failed the PP version on five consecutive sessions, they received the PI&PP version. If subjects were successful ten times within one session, they received one U trial. If they failed the U trial, subjects received three PP trials followed by one U trial in the subsequent test session. If successful in the U trial, subjects received up to nine additional U trials. Testing was continued until subjects were continuously successful in 20 trials within two out of three consecutive sessions. The test ended if they failed the PI&PP version on five consecutive sessions or after not being successful in the U-trials for three consecutive sessions after having received all five PI&PP sessions.

A session consisted of up to 10 trials (depending on task success; if a subject was successful it received up to nine additional trials). Subjects were tested for a maximum of 15 minutes per trial or until success occurred. A trial started as soon as the subject touched a material. In case the subject did not touch any material the trial ended after 15 minutes elapsed. If a subject was successful it received up to nine additional trials, followed by one session of 10 trials on the next testing day. If the subject was not successful during a trial, the session ended. The testing ended when a subject successfully completed 20 U trials within two out of three consecutive sessions.

During testing the experimenter wore mirrored sunglasses, avoided any head movements and was not speaking. Apparatuses were baited out of the subject’s sight. The positions of the food reward and the sides on which the wire and string were placed were semi-randomly balanced across sessions. As soon as the two materials were in position, subjects were allowed to enter the testing compartment.

Follow-up test of the bending task: Subjects that were continuously successful in the bending task were afterwards tested in a follow-up task, in which the basket was fixed on the bottom of the tube with Velcro Fastener. To be still successful, subjects were required to bent the wire at a steeper angle than in the original hook bending task to be able to lift it. Subjects received a minimum of two sessions of ten trials.

### Analysis

All data was HD video recorded and coded in situ as well from the videos. After each session, the final shape of the wire was photographed, the angles measured and the wires stored. The videos were analyzed by using the coding program BORIS^37^. We checked interobserver reliability (13% of the videos were double coded) and found excellent agreement (ICC ≥ 0.801; p < 0.001). In order to maintain comparability to the parrot and corvid studies, we coded and analyzed the same variables as Laumer *et al*. (2017) and Weir & Kacelnik^23^: ‘time until success’ (time from first touch of one of the two materials until reaching the reward), ‘latency between start of the trial until the first modification of the wire’ (latency from subjects entering the test compartment until first modification of the wire occurred), ‘duration of probing with the functional end of the modified tool’ (probing is defined as one end of the wire is inserted into the tube), ‘duration of probing with the unmodified wire’ (probing with the straight wire in the bending task; probing with the v-shaped wire in the unbending task), ‘duration of probing with the modified non-functional tool’, and ‘tool crafting time’ (duration spent modifying the wire) (see electronic supplementary material, section B, Table S2 for descriptions of all variables).

## Results

### Hook-bending task

#### Overview

**Figure 3:**
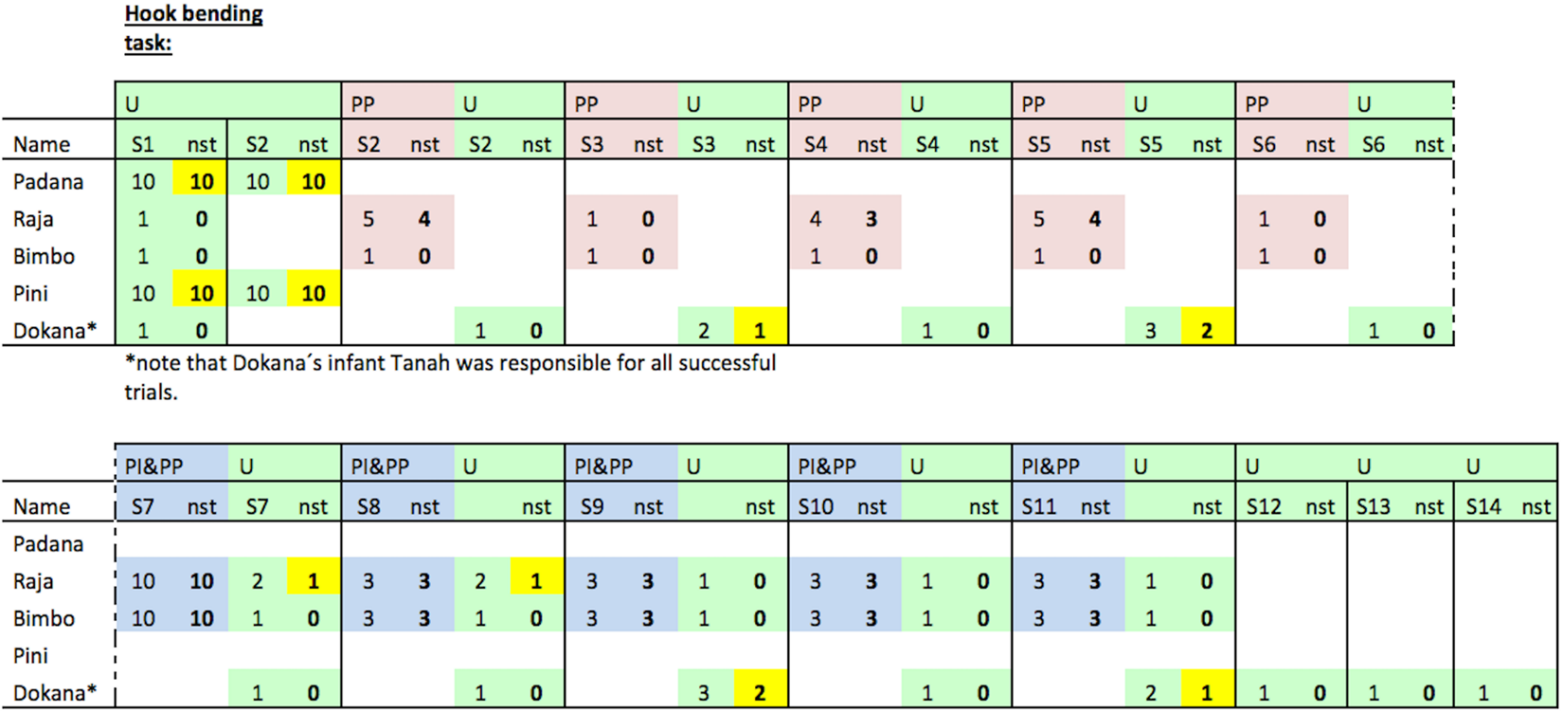
Individual results for each of the versions of the hook bending task. U (=unmodified materials; in green), PP (=pre-prepared materials; in red) and PI&PP trials (pre-inserted and pre-prepared materials; in blue). Numbers indicate the total number of trials per session (S = session) and the numbers on the right side of the respective cell (“nst”) indicate the “number of successful trials” within the respective session (in bold font and marked in yellow if the subject was successful at least once). Note that only subjects that were successful ten times in a row in PP-trials or PI&PP-trials were tested as in U version. Further note that Tanah (Dokana’s offspring that could not be seperated by her mother during testing) was responsible for all successful trials in the hook-bending task after her mother lost interest in the task. Group N (Padana, Raja, Bimbo, Tanah) = non-experienced in the use of hook-tools prior to the experiment; group E (Pini, Dokana) = experienced in the use of hook-tools prior to the experiment.

Two orangutans, one experienced (Pini) and one non-experienced (Padana) in the use of hooks, retrieved the basket from the vertical tube in the first trial with the unmodified materials (U) and continued to do so in subsequent trials (see Fig. 3 for an overview of the results and movie S1 for an example). This meant that they consistently made hook shaped tools out of the straight wire. Two of the remaining subjects were occasionally successful: Raja (non-experienced) successfully retrieved the basket in two out of 8 U-trials after she received a ready-made hook tool was already pre-inserted and pre-bent (PI&PP). Tanah (non-experienced) also succeeded in six out of 20 U-trials whereas her mother (Dokana, experienced) soon lost interest after failing to obtain the food in the first sessions. Bimbo (non-experienced) was also unsuccessful in all of the testing trials.

#### Behavioral description

**Figure 4:**
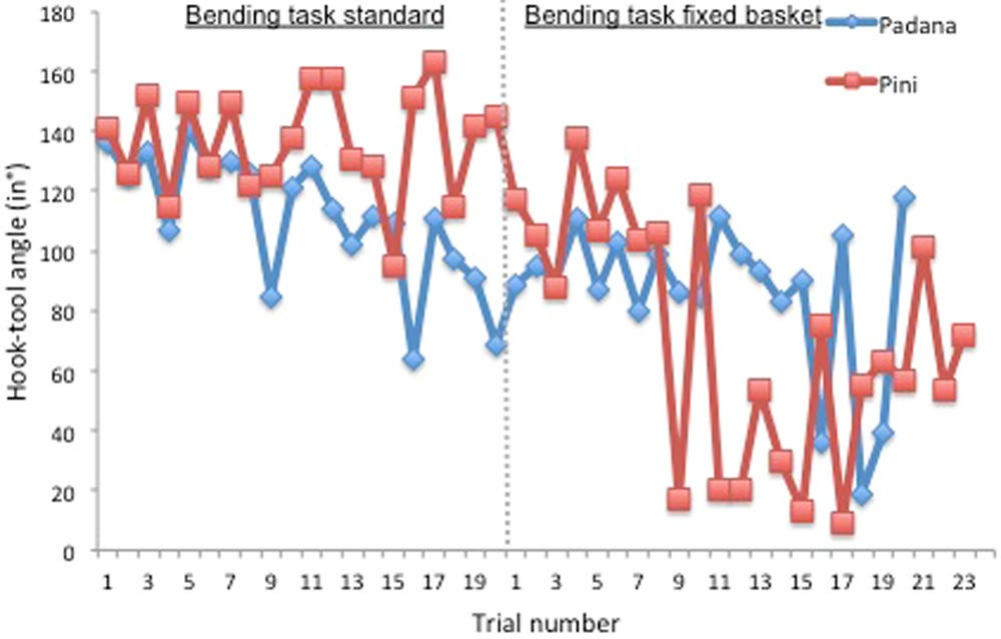
Hook-tool angles (in°) for the bending task standard and the follow-up bending task fixed basket.

**Figure 5:**
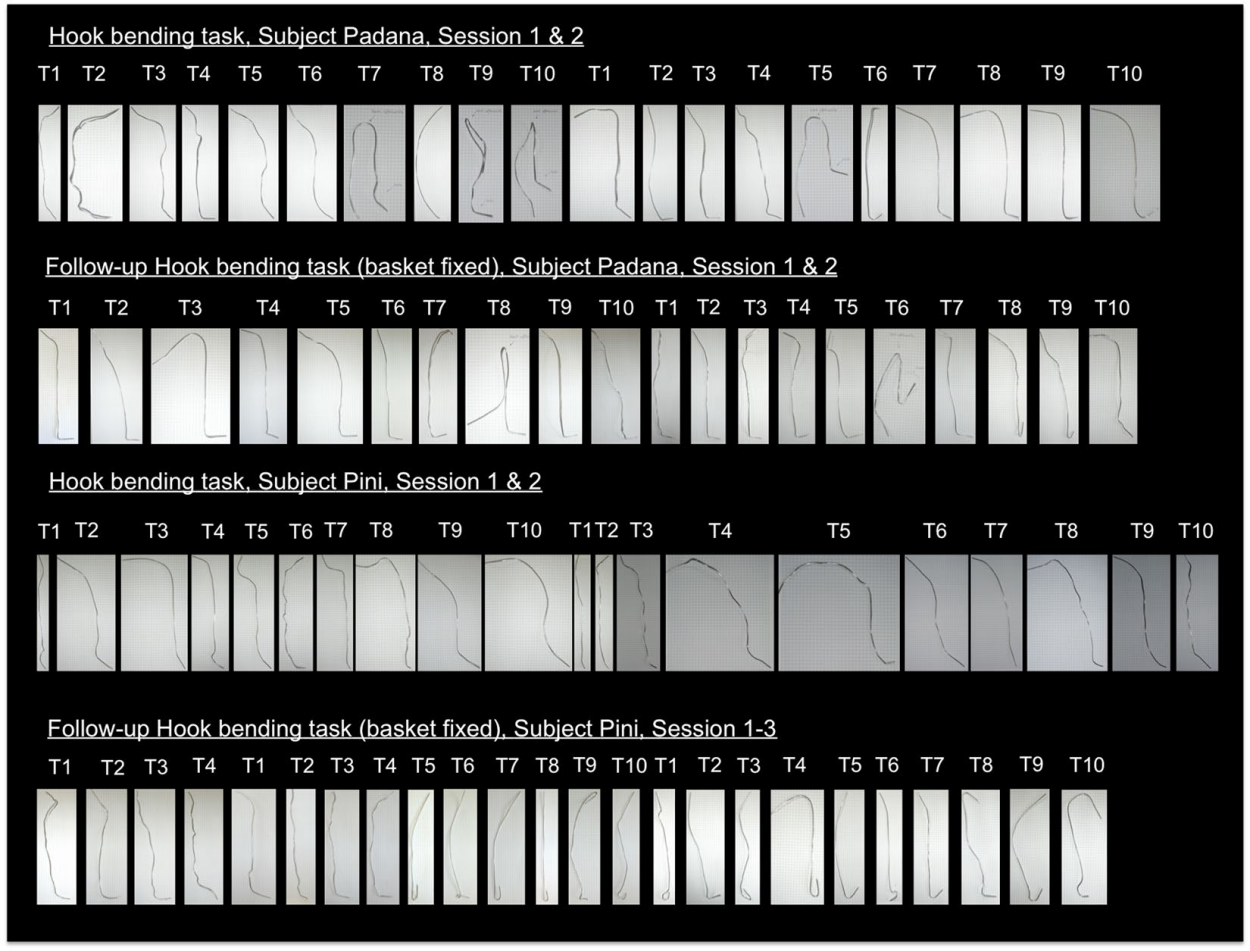
Successful hook tools built by Padana and Pini in the standard hook bending task and the follow-up task with the basket fixed on the bottom of the tube (T = Trial). Hook tools are depicted with the working end facing downwards. After using the tool successfully, Padana kinked the tool in six trials in the middle section when handing it over to the experimenter through the bars of the testing compartment (see HB task: Session 1: T7, T9 & 10, Session 2: T5; Follow-up HB task: Session 1: T8, Session 2: T6). During that the previously fashioned hooks were not damaged.

Padana took five minutes and 15 seconds to retrieve the basket in her first trial: She immediately chose the wire and inserted it into the tube. After initially probing with the unmodified wire, she bent the tip of the tool by using her mouth/teeth while holding the wire with one hand. During the course of the trial she modified the tool using this technique a total of three times, straightening the rest of the tool two times and always inserting the tool in correct orientation. The rest of the time she kept probing with the tool after she finally succeeded in hooking the handle and retrieving the basket (see movie S2). Both the time time until success and the tool crafting time decreased over the course of the trials (see SI, section c, Fig. S1). The probing time with the modified functional and non-functional tools tended to decrease when comparing the first ten to the last ten trials (see SI, Fig. S1). Additionally, the hook angles were steeper angle when comparing the first to the last ten trials (mean_first ten trials_ = 123.2**°** ± 15.4**°**, mean_last ten trials_ = 99.7**°** ± 19.2**°**; see Figs 4 and 5). Further the duration between the start of the trial until the first modification of the wire decreased. Directly after the start of the trial she picked up the wire immediately (mean = 2.1 sec) and inserted it. In only two out of a total of 20 trials the string was touched (total durations: 7.5 s and 1.5 s) and only once inserted. She lifted the basket by pulling it vertically up. She used the tip of the previously bent functional hook-tool and the sidewall of the tube to retrieve the basket only once during the first ten trials (in trial 8). She never inserted any of the two materials into the unbaited tube. To manufacture the tool, she showed a variety of bending techniques: she mainly used her teeth to bend one end of the wire while holding it with her hand and then inserted the modified end. In six occasions, she also modified the wire by using both hands. Only once she bent the wire over the rim of the tube with her hand while the distal end was inserted.

Pini was successful for the first time in her first trial after one minute and 44 seconds. She immediately bent a straight wire into a hook with a steep angle three seconds after she picked up the wire and directly afterwards inserted it in correct orientation. She modified it a total of five times (until finally succeeding in retrieving the basket) by using her mouth and teeth while holding it in one hand and immediately inserted it after manufacture in the correct orientation (see movie S3, for a detailed description of this video see SI).

When comparing the first ten to the last ten trials, there was no improvement in the time until success, in the duration of probing with the unmodified wire, in the duration of probing with the modified but non-functional wire, in the tool crafting time, nor in the bent angles of the hooks (mean_first ten trials_ = 134.7**°** ± 12.6**°**, mean_last ten trials_ = 138.6**°** ± 20.5**°**; see Figs 4, 5 and SI, section d, Fig. S2). Further the duration from the start until the first modification of the wire (mean 54.7 ± 54.4 s, median = 31.9 s), as well as the probing time with the functional tool increased (see SI, section d, Fig. S2). Pini picked up the wire directly after the start of the trial (mean = 8.5 ± 8.6 sec) and inserted it. The string was touched in six out of a total of 20 trials, was manipulated on average for 9 ± 15.9 seconds and never inserted in any of the tubes. During six out of the first ten trials she used the previously bent hook-tool and the side of the tube’s wall in order to lift the basket, in four trials (trial 2, 4, 6 & 9) she pulled the basket up vertically. To bend the wire, she mainly bit into one end of the wire while holding it in her hand and then inserted the modified end. Only in two occasions she bent the wire over the rim of the tube with her hand while the distal end was inserted.

During the PP-trials of the hook-bending task (pre-bent hook tool was provided), Bimbo was not successful. He immediately picked up the wire, but inserted the pre-bent wire in the wrong orientation in the first three out of the five PP*-*trials. Although the hook was hooked into the handle in trial five, he did not pull the basket up and then soon lost interest. Raja succeeded in 11 out of 16 PP*-*trials but not consistently for 10 trials in a row. She immediately picked up the ready-made hook tool, but inserted it in only half of the trials in the correct orientation. Interestingly she sometimes modified the hook with her teeth, straightening the curvature slightly. Only after receiving PI&PP-trials (hook tool pre-inserted, pre-bent and handle hooked), both subjects continuously lifted the basket up.

After receiving all scaffolding steps, Raja was successful in two out of 8 U-trials. Although she immediately picked up the wire in both trials it took her between 30 and 60 seconds until she started to modify it. Although her probing duration with the modified functional tool in these two trials increased, it took her between seven and ten minutes to retrieve the basket (see SI, section e, Fig. S10). Raja manipulated the string only in one of the two trials for 37 seconds and never inserted it. She modified the wire by bending it over the rim of the tube and by oral manipulation, once she straightened it by using both hands.

The six-year-old Tanah was successful in six out of a total of 20 U-trials. Although there was an increase in her probing time with the modified functional tool, there was no progress in terms of overall success time, it took her within eight to 13 minutes to pull up the basket (see SI, section e, Fig. S11). In the majority of these trials she started to modify the wire after between three and seven minutes. Tanah manipulated the string in all of the six trials and inserted it in five of the trials. She never inserted any of the materials in the unbaited tube. Bending of the wire occurred mostly through bending the wire over the rim of the tube, by pushing it into the tube and by oral manipulation.

#### Follow-up test

Both orangutans were still able to retrieve the basket from the first trial (Pini was not successful in her fourth trial, but from the next testing day on she retrieved the basket continuously) despite the fact that the basket was attached to the bottom of the tube with Velcro. More importantly, both orangutans fashioned hooks with a steeper angle than in the standard hook bending task (Padana: mean_standard HB task_ = 111.45**°** ± 21**°**, mean_HB2-fixed basket_ = 85.95**°** ± 25.2**°**, median_standard HB task_ = 113**°**, median_HB2-fixed basket_ = 90**°**; Pini: mean_standard HB task_ = 136.7**°** ± 17**°**, mean_HB2-fixed basket_ = 68.7**°** ± 41.2**°**, median_standard HB task_ = 139.5**°**, median_HB2-fixed basket_ = 67.5**°**; see Figs 4, 5).

Padana was continuously successful. Directly after the start of the trial she picked up the wire immediately (mean = 2.8 ± 1.1 s), modified (mean_latency between start of trial until first modification_ = 4.3 ± 1.1 s) and inserted it. She spent the majority of time probing with the functional tool (see SI, section d, Fig. S3) and never inserted the unmodified wire (in the cases probing was coded as „probing with modified but non-functional tool“ (see SI, section c), the angle of the hook-tool was not bent steep enough to pull the basket up). Hooks were bent at a steeper angle when comparing the first ten to the last ten trials (mean_first ten trials_ = 92.5**°** ± 9**°**, mean_last ten trials_ = 79.4**°** ± 33.3**°**; see Figs 4, 5). She never touched the string or inserted a material into the unbaited tube. Padana used the same bending technique during all trials: she bent one end of the wire by using her teeth while holding it with her hand and then inserted the functional end. She got faster (mean time until success = 71.4 ± 78.6 s) compared to the standard hook bending task (mean = 128.3 ± 112.1 s).

Pini picked the wire up immediately (mean = 3.6 ± 1.5 s), modified (mean_latency between start of trial until first modification_ = 6.7 ± 3.4 s) and inserted it. She spent the majority of time probing with the modified functional tool, she rarely probed with the modified but non-functional wire (only in the first seven trials she inserted the modified wire while it was still not fully functional) and never inserted the non-modified wire (see SI, section d, Fig. S4). Nevertheless, from the 8th trial on, Pini immediately bent the wire at the required functional angle and inserted it. Further, from this trial on, the time until success as well as the duration of probing with the functional tool rapidly decreased (see SI, Fig. S4). Furthermore, from the eighth trial on, hooks were bent at a steeper angle (mean _first eight trials_ = 111.1**°** ± 14.1**°**, mean _last trials_ = 47.44**°** ± 33**°**; see Figs 4, 5). Compared to the standard hook bending task she got faster (mean time until success = 128 ± 181 s; standard task: 271 ± 177 s; median „time until success“_fixed basket_ = 53 s; median „time until success“_standard task_ = 303 s). Pini never picked up the string (only once touched it for 2.4 s) nor inserted any of the materials in the unbaited tube. As in the previous standard hook bending task she mainly bit into one end of the wire while holding it in her hand and then inserted the modified end. In two occasions, she bent the wire directly inside the tube with her fingers.

### Unbending task

#### Overview

**Figure 6:**
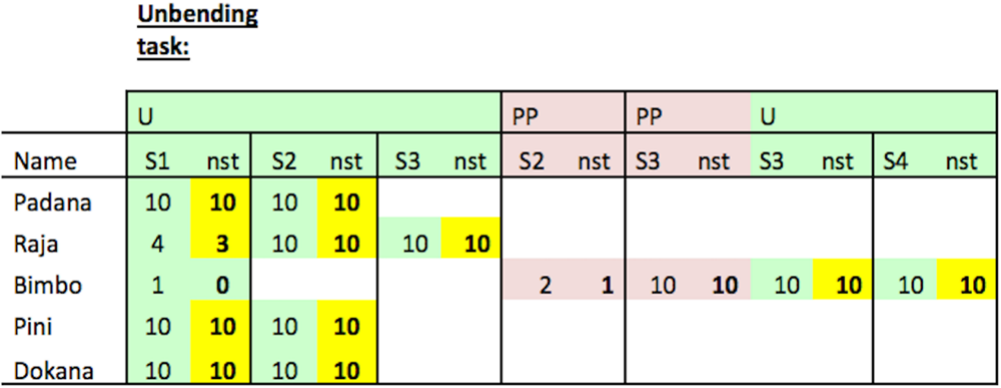
Individual results for each of the versions of the unbending hook task. U (=unmodified materials; in green), PP (=pre-prepared materials; in red). Numbers indicate the total number of trials per session (S = session) and the numbers on the right side of the respective cell (“nst”) indicate the “number of successful trials” within the respective session (in bold font and marked in yellow if the subject was successful at least once). Note that only subjects that were successful ten times in a row in PP-trials were tested as in U version. Group N (Padana, Raja, Bimbo) = non-experienced in the use of hook-tools prior to the experiment; group E (Pini, Dokana) = experienced in the use of hook-tools prior to the experiment.

**Figure 7:**
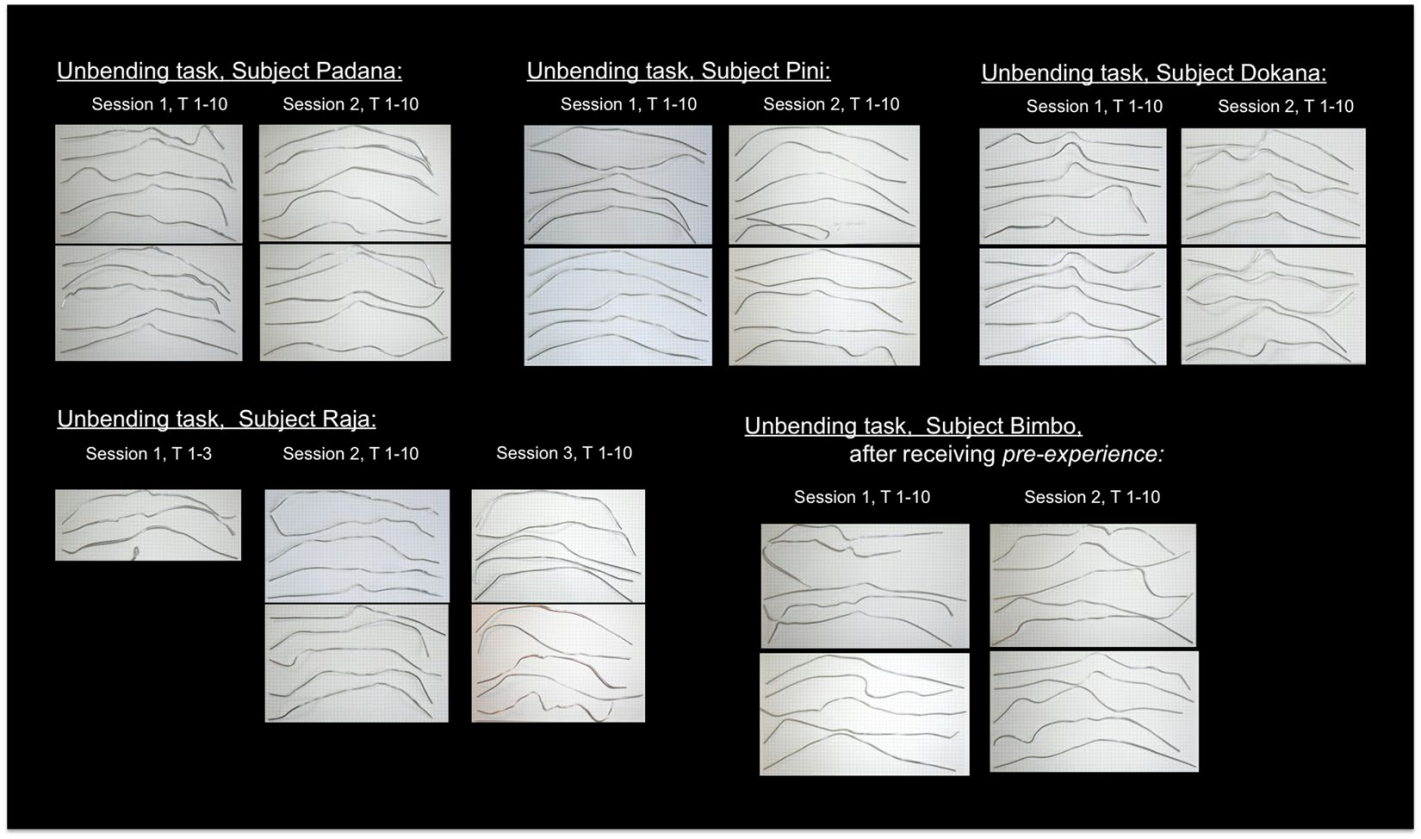
Successful tools unbent by Padana, Pini, Dokana, Raja and Bimbo (T = Trial). After using the tool successfully, Pini and Bimbo kinked the tool in one trial in the middle section when handing it over to the experimenter through the bars of the testing compartment (Pini: Session 2, Trial 5; Bimbo: Session 1, Trial 2).

All subjects except Bimbo successfully unbent the wire, pushed the reward out of the horizontal tube in the first U-trial and continued to do so in subsequent trials (see Fig. 6 for an overview, Fig. 7 for an overview of the successful tools). Bimbo consistently succeeded after receiving PP-trials (wire already straightened; see Fig. 6).

#### Behavioural descriptions of the unbending task

Padana was successful within 29 seconds in the very first trial. She immediately picked up the wire after the start of the trial (mean of all trials = 2.9 ± 1.2 s) and unbent it (mean_latency between start of trial until first modification_ = 5.5 ± 2.1 s). For probing she always used the modified functional tool and never probed with an unmodified wire nor with a modified but non-functional wire (see SI, section d, Fig. S5). The string was manipulated only once during the entire testing period for 12 seconds and none of the materials were inserted in the unbaited tube. She used several techniques and often a combination of them to unbent the wire including unbending by using both hands, by biting into one end of the wire and simultaneously pulling with one hand and by pushing the bent wire into the tube and then unbending it directly in the tube by using one hand.

Pini was successful within 27 seconds in her very first trial. She immediately picked up the wire after the start of the trial (mean of all trials = 3.2 ± 0.7 s) and unbent it (mean_latency between start of trial until first modification_ = 5.3 ± 1.1 s). She always used the modified functional tool for probing (see SI, section d, Fig. S6). She never touched the string during the entire test and none of the materials were inserted in the unbaited tube. To unbent the wire, Pini mainly bit into one end of the wire and simultaneously pulled with one hand. She rarely unbent the wire by pushing the bent wire into the tube and then unbending it directly in the tube by using one hand and only once used both hands to straighten it.

Dokana was successful within 52 seconds in the very first trial. She immediately picked up the wire after the start of the trial (mean of all trials = 3.6 ± 2.5 s) and unbent it (mean_latency between start of trial until first modification_ = 9.3 ± 3.5 s). For probing she always used the modified functional tool and never probed with an unmodified wire nor with a modified but non-functional wire (see SI, section d, Fig. S7). The string was manipulated only in the first trial for six seconds and none of the materials were inserted in the unbaited tube. She mainly unbent the wire inside the tube by pushing with the fingers. Only in three occasions she unbent it by pulling with both hands.

It took Raja more than nine minutes to successfully retrieve the reward during her very first trial. She was not successful in her fourth trial, but from the next testing day on she retrieved the food continuously. Her “time until success” decreased, as well as the “tool crafting time” (see SI, section d, Fig. S8). Raja rarely probed with the modified but non-functional wire (only in her first trial for 56 seconds and in her third trial for three seconds and never probed with the unmodified wire. In her first three trials she manipulated the string (for a total of 26 s, 8 s and 18 s) and in the first two trials she also inserted the string into the baited tube. She inserted the wire in four trials into the unbaited tube (in Trial 4 for 180 s and in Trial 5, 16, 21 for under seven seconds). Raja picked up the wire after the start of the trial after a mean of 9.6 ± 24.1 s and modified it (mean_latency between start of trial until first modification_ = 12.6 ± 24.4 s). She unbent the wire usually by straightening it with both hands or by biting into one end and pulling with one hand at the other end of the wire. Only in trial 2 & 3 she unbent the wire inside the tube.

Bimbo was not successful in his first U-trial. After receiving PP-trials (a straight piece of wire was placed next to the apparatus), Bimbo was one time successful in session 1 and continuously successful from session 2 on. The first time it took him 54 s to successfully unbent the wire and retrieve the food. Thereafter he was continuously successful (see SI, section d, Fig. S9). He rarely probed with the modified but non-functional wire (only in 4 out of 20 trials for a mean duration of 23 ± 7.8 s) and never probed with a non-modified wire. Bimbo picked up the wire directly after the start of the trial (mean duration 3.1 ± 0.6 s) and modified it (mean_latency between start of trial until first modification_ = 10 ± 3.2 s). He never touched the string or inserted any of the materials in the unbaited tube. He mainly unbent the wire inside the tube by pushing with the fingers, in four occasions he unbent the wire by pulling with both hands on each side of it.

## Discussion

Getting back to the first two aims of present study, we are hereby able to show that orangutans can spontaneously innovate a hook-tool out of a novel pliant material to solve a novel problem. Two out of the five subjects (Padana and Pini) were continuously successful from their very first naïve U-trial onwards (straight wire and string were placed next to the apparatus). Furthermore, both subjects improved their tools’ design when a stronger angle was required to lift the basket out of the tube due to a stronger fixation to the tube floor. In the unbending task, four out of the five orangutans spontaneously unbent the wire from their very first naïve U-trial onwards (v-shaped wire and string were placed next to the apparatus). The remaining subject, the adult male Bimbo, was continuously successful after receiving PP-trials (provided wire was already straightened). Similar to Goffin’s cockatoos^13^ and New Caledonian crows^23^, two orangutans were able to make different tool types out of the same material, as they were consistently successful in both, the bending and the unbending task.

In the bending task the two adult females, Padana and Pini, spontaneously bent hook tools out of straight wire and retrieved the basket within the first two (Pini) and six minutes (Padana) of their very first trial (note that the New Caledonian crow Betty was the first time successful in her second trial, three of the four rooks on their first trial, one on the fourth trial (but note that both corvids had received pre-experience with ready-made hook tools in the same context and same apparatus beforehand) and the two non-habitually tool using Goffin’s cockatoos in their ninth and tenth test trial (one after having received pre-experience)^9,12,13^. In the very first trial both orangutans chose and inserted the wire within the first 30 seconds. Pini even bent the tip of the wire by using her mouth/teeth in a steep angle three seconds after picking up the wire for the first time (see movie S3) and Padana bent a hook directly after probing with the unmodified wire (see movie S2). Both subjects modified the tip of the tool several times. Padana also straightened the rest of the tool twice and both always inserted the tool in correct orientation and spent the rest of the time probing with the tool. Therefore, it seems unlikely that they learned to create a hook tool by trial and error, since the actions performed by both orangutans seemed quite spontaneous and goal directed. The corvid’s pre-experience with the task, the ecological pre-dispositions and foraging adaptions^10^ may have influenced their innovation speed. In case of the orangutans, their morphology (the habitual coordinated use of the hands and mouth for tool-manufacture is likely to allow them to execute more precise bending and probing manipulations than a beak and claws) and the fact that, like all great apes species they are habitually constructing sleeping nests^38^ could have facilitated the fast innovative process. In order to create the basic layer of the nest, large branches are pulled, bent at the base and broken towards the centre in order to lock them by weaving and twisting motions, usually followed by adding smaller branches in the same style or by detaching them completely and placing them on top^39^. Nevertheless, the hook-tool manufacture still required ‘bending actions’ in a novel context, using a novel material and using different techniques than described during nest-building (using the teeth to bend over the end of the wire while holding it in one hand, bending while holding the by using both hands using, bending the wire over the rim of the tube afterwards inserting it correctly), which thereby constitutes a case of innovative tool manufacture.

Both orangutans were continuously successful throughout all following test trials. In contrast to Pini who showed no improvement in terms of overall efficiency in the course of the trials (note that it took her only two minutes to lift the basket in the very first trial), Padana’s time until success and her probing time with the modified tool decreased. This is likely due her improvement of the tools’ designs, since the angles of the tools were bent at an approximately 20**°** steeper angle when comparing the first ten to the last ten trials. Pini never tried to insert the distractor material (string) in any of the test trials, Padana inserted the string only once, but both orangutans inserted the modified wire within the first few seconds directly after the start of the trial. Furthermore, subjects spent more time probing with the functional modified tool, than with the modified but non-functional or with the non-modified wire. Both orangutans used different bending techniques across trials (e.g. either using the teeth to bend one end while holding it with one hand, bending by using both hands, bending the wire over the rim of the tube afterwards inserting it correctly), which indicates that their success was not a result of a learned chain of manipulative actions^23^. Furthermore, since both orangutans were immediately continuously successful within the short time frame of the first trial, it is unlikely that random interactions followed by associative learning would have been sufficient to succeed in this task. To be successful several unrewarded steps must be executed in a specific order^1^: bending only one end of the wire while keeping the rest of it straight (2) producing a hook whose angle is functional^3^ inserting it in correct orientation^4^ pulling only when the basket’s handle is hooked and^5^ pulling until the basket is within reach.

In the unbending task Padana, Pini and Dokana were successful within less than one minute in their very first trial, Raja within less than ten minutes. The fact that almost all subjects were able to solve the task immediately and continuously, hardly ever touched or inserted the string and modified the wire directly within the first seconds of the trial, strongly indicates that their actions were executed in a goal-directed way. Additionally, as in the bending task, individuals showed different techniques for unbending the wire including: bi-manual coodination, biting into one end of the wire while pulling with one hand, or pushing the bent wire into the tube and then unbending it inside the tube. Similar to children^16^, the success rate was higher in the unbending task compared to the hook bending task. This is not surprising since, unlike the hook-bending task, which requires several unrewarded steps, the unbending task requires only unbending and inserting of the tool.

The third aim of this study was to investigate the effect of pre-experience with hooked tools by providing stepwise scaffolding experience to half of the subjects after initial failure. Pre-experience with ready-made hook tools had no effect on subject’s overall success in the hook bending task, although in children using a pre-made tool led to higher task success^19^. In contrast to a Goffin’s cockatoo that used the provided ready-made hook-tool in correct orientation early on^13^, both orangutans inserted it in only half of the trials with the hook at the working end. Although Raja and Bimbo (non-experienced in using hook-tools prior to the experiment) were finally continuously able to retrieve the basket with the pre-inserted hook-tool, they were very rarely (Raja in only two trials) or never successful at all in the hook bending task (Bimbo). Furthermore, previous experience with the unbending task seems to have no influence on subject’s performance in the bending task, since none of the subjects that received the unbending task first became consistently successful. This is not surprising since different motor actions and stimuli configurations were necessary to solve the two tasks. The six-year-old Tanah was able to retrieve the basket in six trials, but showed no improvement in latency to success. Further she mostly started to modify the wire after several minutes had passed, indicating that she failed to associate a modified wire with task success. In contrast to the two continuously successful subjects, Tanah’s tool modification seemed to occur in a rather uncoordinated manner by bending the wire over the rim of the tube, by pushing it into the tube or by oral manipulation. Moreover, in the majority of trials she manipulated the distractor material and inserted it.

Other than Goffin cockatoos, the other two species tested so far had seen or used a hook tool in the same context and with the same apparatus before^9,12,13^. In case of the rooks it was suggested that they had invented a technique to create a ‘known’ tool (a tool that they had already used in the same context; although hereditary predispositions from nest-building can also not be fully excluded), but it cannot be confirmed that they invented a novel tool itself. While it is highly likely that children have encountered hooks before, it is unlikely that they had previously used a pipecleaner in the context of retrieving a basket out of a vertical tube^15^. Although four of our subjects had *experience* using a raking tool in a study that was published eight years before (^35^; see SI, chapter 1), that tool (a wooden rake whose head was 30 × 12 × 1 cm in size) differed substantially in its physical appearance from the one used in the current study. Moreover, subjects had used it in another context and physical orientation (to rake a reward resting on a horizontal trap-table; at the beginning of each trial the rake was pre-positioned with the handle reaching through the wire mesh, which allowed only restricted movement of the rake possibly leading to a limited information gain about the functionality of the tool). Moreover, only two out of the four subjects that had participated in this study manufactured hook tools. It can thus be assumed that this raking-experience was not greatly relevant for task-success. One of the two immediately successful subjects (Pini) had used a rigid unbendable metal hook-tool in order to pull in a plastic bottle filled with juice hanging from the ceiling in front of the test compartment in a study that was conducted ten years before^36^. It cannot be excluded that this experience could have influenced Pini’s performance in the present study. Although it cannot be confirmed that she invented a novel tool itself, nevertheless she invented a novel technique to create a possibly known tool to solve a novel problem. Further Dokana, that had received the same raking-experience as Pini, was never successful. Since Padana successfully bent hooks and retrieved the basket from her first trial on without having this kind of raking experience, it seems not to be essential for task success. Additionally, subjects Raja and Tanah, who had no raking-experience with hooked raking tools beforehand, successfully manufactured hook tools and retrieved the basket (Raja in two trials, Tanah in six trials).

In the unbending task, all but one subject were continuously successful from their first trial on. The adult male Bimbo was continuously successful from Session 2 on after receiving the first scaffolding step (straight tool was provided next to the apparatus). It took him less than a minute to unbent the wire and get the reward. If success was due to the number of exposures to this task or receiving pre-experience, or a mixture of both cannot be determined.

The fourth aim of this study was to investigate whether successful subjects were able to improve the hook-design feature. The corvid’s hook tools were mostly bent at relatively wide angles^9,12,23^ and were bent more centred than the Goffin cockatoo’s hook tools^13^. Tools bent at wider angles were better for the rooks since they could only reach to a certain depth before running out of wire and usually pulled the basket up diagonally^12^. Betty mostly bent their hook tools by wedging the tip of the wire outside of the tube and then by pulling sideways and the rooks bent the tool by bending it over the rim of the tube and then turning it around^9,12^. Goffin cockatoos bent tools at steeper angles, probably due to different beak morphology which allowed them to directly bent the tool in their beak by pressing the upper mandible against the rounded corner of the lower mandible^13^. As the basket was fixed to the bottom of the tube with Velcro fastener, which required the crafting of a steeper hook angle to be able to retrieve the basket, both orangutans bent the wire at a steeper angle compared to the previous standard hook-bending task. Again, both subjects immediately picked up, modified and inserted the wire and never manipulated nor inserted the string. Padana’s tools were bent as well at a slightly steeper angle when comparing the first to the last ten trials, although this did not seem to have an impact on her speed of solving the task. Interestingly Pini’s latency to success as well as the duration of probing with the functional tool decreased rapidly from her eighth trial onwards. Looking at the actual tools, it is very likely that this might be due to improved tool design since fashioned hooks were bent at an even steeper angle from the eighth trial onwards. In contrast to Padana, which lifted the basket by pulling it vertically up in the original hook-bending task, Pini mostly used the previously bent tip of the tool and the sidewall of the tube to lift the unattached basket. Since she became faster from trial eight onward, it is likely that Pini learned by trial and error to bend steep enough angles in the fixed basket condition.

As mentioned previously, hook bending was suggested to represent an “ill-structured problem”^15,16^, since the final goal has to be kept in mind when exercising all the necessary steps to achieve it (steps include bending the wire at a functional angle while keeping the other part of it straight, inserting it in correct orientation, hooking the handle of the basket, pulling it up until the basket is within reach). Other studies further confirm that children gain the majority of their tool use behaviours by social learning and employ innovation as a last resort^40^. In contrast to the obvious difficulties younger children face when confronted with the hook-bending task, two orangutans were able to continuously solve the problem from their very first trial on within only two and six minutes. Further Padana’s and Pini’s actions seemed to be executed in a goal directed way. This finding supports the assumption that they used and coordinated relevant pieces of information targeted as a solution to the problem. Nevertheless, only two out of the five orangutans solved the hook-bending problem, although they were given all relevant information in order to solve the task prior to the actual experiment (experience that a wire retains its form after bending manipulations, basket can be pulled up by pulling on the handle). Interestingly, when looking at subject’s performance in other tool use tasks (for details see SI, section f, Table S3), the two most successful orangutans in the hook-bending task also obtained high rankings in terms of tool proficiency compared to the other subjects (Pini was ranked number 1 and Padana number 3). It is assumed that the main obstacle for young children is retrieving the required knowledge from memory^17^. Taken together, in order to be successful, most likely a mixture of advanced modes of cognitive processing (e.g. high behavioural flexibility, fast individual learning, precise sensorimotor control) that lead to an integration of accessing, retrieving and coordinating knowledge, seem to be the key to innovate a successful solution to the hook-bending problem. Nevertheless, in order to get a more detailed look on the emergence of the ability to solve an ill-structured tool problem, which apparently could be seen as a milestone in some specie’s cognitive development^41^, further studies that test the standardized hook-bending task in other habitual tool users, such as e.g. the chimpanzee or capuchin monkey, are required. Moreover, to shed light on possible parallels in the ontogeny of the mechanisms underlying tool-making innovations in primates, it would further be necessary to test different age classes in tasks such as the hook-bending paradigm.

## Electronic supplementary material

Movie S1

Movie S2

Movie S3

Supplementary Information

Supplementary Dataset
