# Supplementary material for: Spontaneous innovation of hook-bending and unbending in orangutans (Pongo abelii)
Source: Sci Rep. 2018 Nov 8;8:16518. doi: 10.1038/s41598-018-34607-0 (PMC6224503; doi:10.1038/s41598-018-34607-0)
Supplement: Supplementary file 4 — Supplementary Information [file 41598_2018_34607_MOESM4_ESM.pdf]

Supplementary Information

TITLE: Spontaneous innovation of hook-bending and unbending in orangutans (*Pongo abelii*)

AUTHORS: Laumer, I. B., Call, J., Bugnyar, T. Auersperg, A. M. I.

Movie S1: Video of the bending and unbending task.

Movie S2: Video of Padana´s first trial of the bending task. Padana immediately chose the wire and inserted it. After probing with the unmodified wire she bent the tip of the tool by using her mouth/teeth while holding the other end in her hand. During the course of the trial she modified the tool using this technique a total of three times, straightened the rest of the tool two times and always inserted the tool in correct orientation. The rest of the time she was constantly probing with the tool after finally being successful in hooking the handle and retrieving the basket.

Movie S3: Video of Pini´s first trial of the bending task. She immediately bent a hook with a steep angle out of the straight wire within the first seconds. She modified it a total of five times until finally retrieving the basket by using her mouth and teeth and immediately inserted the tool after manufacture in correct orientation. She even combined this actions by trying out another technique that she used in the floating peanut experiment that was conducted ca. nine years before: In this experiment subjects could reach the peanut by raising the water-level by spitting water into the tube. Notably, the first action Pini performed was to try to suck water out of the water dispenser (in the video you can see that she is reaching down with her head; the water dispenser is located at the bottom on the left side of the testing window). Since we turned off the water prior to each test trial (and as designing the tubes made sure that there are holes at the bottom of each tube) she was not able to spit water into the tube. Interestingly she then peed into the baited tube (highly likely because of the lack of water) after having modified and inserted the hook tool a fourth time.

a) Subject information

Table S1 Names, division into two testing groups (E= experience group; C= control group), sex, date of birth and experimental history with raking tools of the six orangutans. (1=Mulcahy & Call, 2006; 2=Girndt et al., 2008; 3=Martin-Ordas et al., 2008; 4= Mulcahy & Call, 2006)

Group	Name	Sex	Date of birth	Rearing	Experience with raking
A	Padana	female	18.11.1997	mother reared	2, 3
A	Raja	female	26.09.2003	mother reared	no experience
A	Bimbo	male	20.09.1980	nursery reared	1, 2, 3
B	Pini	female	30.06.1988	mother reared	1, 2, 3, 4
B	Dokana	female	31.01.1989	mother reared	1, 2, 3, 4
B	Tanah	female	16.06.2009	mother Dokana	no experience

Supplementary References (for reference 2 and 4 see main manuscript):

1) Mulcahy , N.J. & Call, J. (2006). How great apes perform on a modified trap-tube task. *Animal Cognition*, 9, 193-199.

3) Martin-Ordas, G., Call, J., & Colmenares, F. (2008). Tubes, tables and traps: great apes solve two functionally-equivalent trap tasks but show no evidence of transfer across tasks. *Animal Cognition*, 11, 423–430.

Detailed description of experimental experience with raking tools

Mulcahy & Call (2006) used horizontal trap-tubes and as a tool a straight wooden dowel (diametre 1.5cm, lenghth 120cm). Martin-Ordas and colleagues (2008) used a horizontal trap-tube and a trap-table. The tool was in both conditions a straight wooden dowel (diametre 0.5cm; length 50cm). In the study by Girndt and colleagues (2008) a horizontal trap-table was used. The head of the rake was made out of wood (30x12x1cm) and was attached to a wooden rod (diametre 2cm, length 44 cm). In the study by Mulcahy & Call (2006) two subjects used a rigid unbendable metal hook (35cm in length) to rake a plastic bottle filled with juice hanging from the ceiling outside the enclosure into reach.

Additional housing information

Subjects were housed in a large, enriched outdoor and indoor area at the Wolfgang Köhler Primate Research Centre (WKPRC) located in Leipzig Zoo (Leipzig, Germany). The enclosures were equipped with naturally climbing structures and various enrichment items. Food and water were available ad libitum. During the three main meals, the orangutans

received fresh fruits, vegetables, eggs, cereals, leaves and sometimes meat. All apes participate regularly in non-invasive cognitive studies. Animal husbandry and research comply with the EAZA minimum standards for the accommodation and care of animals in zoos and aquaria and the WAZA ethical guidelines for the conduct of research on animals by zoos and aquariums. The research adhered to all German laws regarding animal testing and holding. All orangutans participated on a voluntary basis.

b) *Supplementary procedures*

Table S2 List of variables measured in analysis

Variables measured
Time until success (excluding time when subject was not interacting with material/apparatus)
Duration of probing with unmodified wire (probing is defined as one end of wire is inserted into the tube)
Duration of probing with modified non-functional wire
Duration of probing with modified functional tool
Latency between start of the trial and first touch of the wire
Latency between start of the trial and first modification of the wire
Tool crafting time (duration spent modifying the wire)
Proximal, middle or distal modification
Place of modification (at apparatus or elsewhere)
Functional or non-functional end of modified tool first used to probe
Technique used to modify wire
Wire turned around and how often
Final shape of tool
String touched
Duration of string manipulation
String inserted
Duration of string insertion
String or wire inserted in unbaited tube
Duration of insertion in unbaited tube

c) Descriptive results of successful trials in the hook-bending task

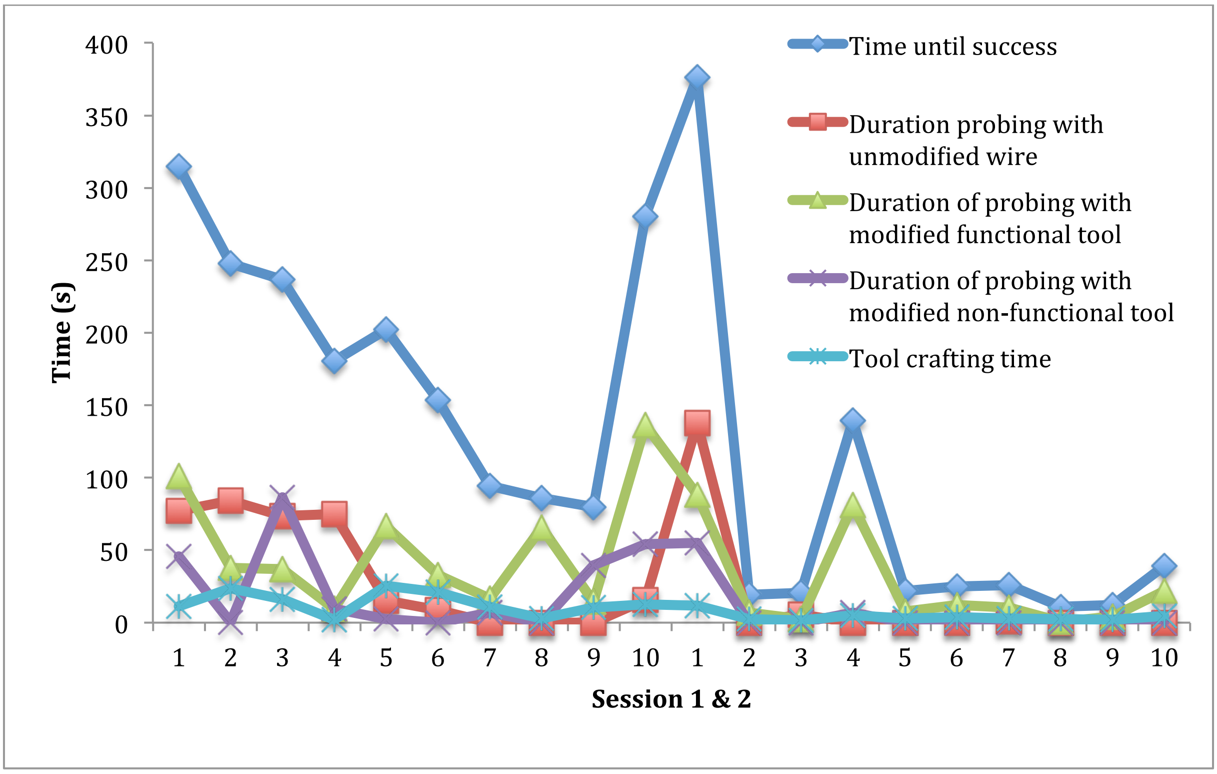

Fig. S1 Results of Padana´s successful trials in the hook-bending task.

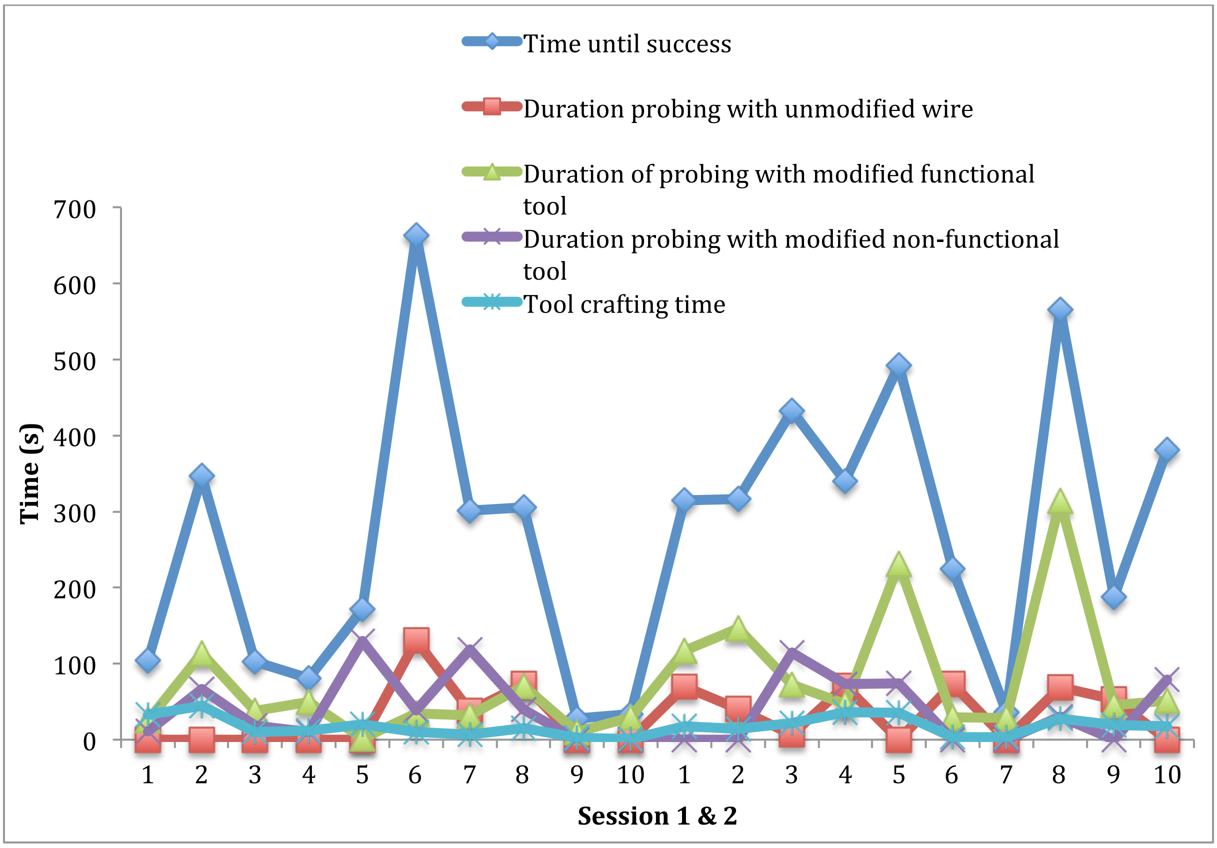

Fig. S2 Results of Pini´s successful trials in the hook-bending task.

Descriptive results of successful trials in the hook-bending task with fixed basket

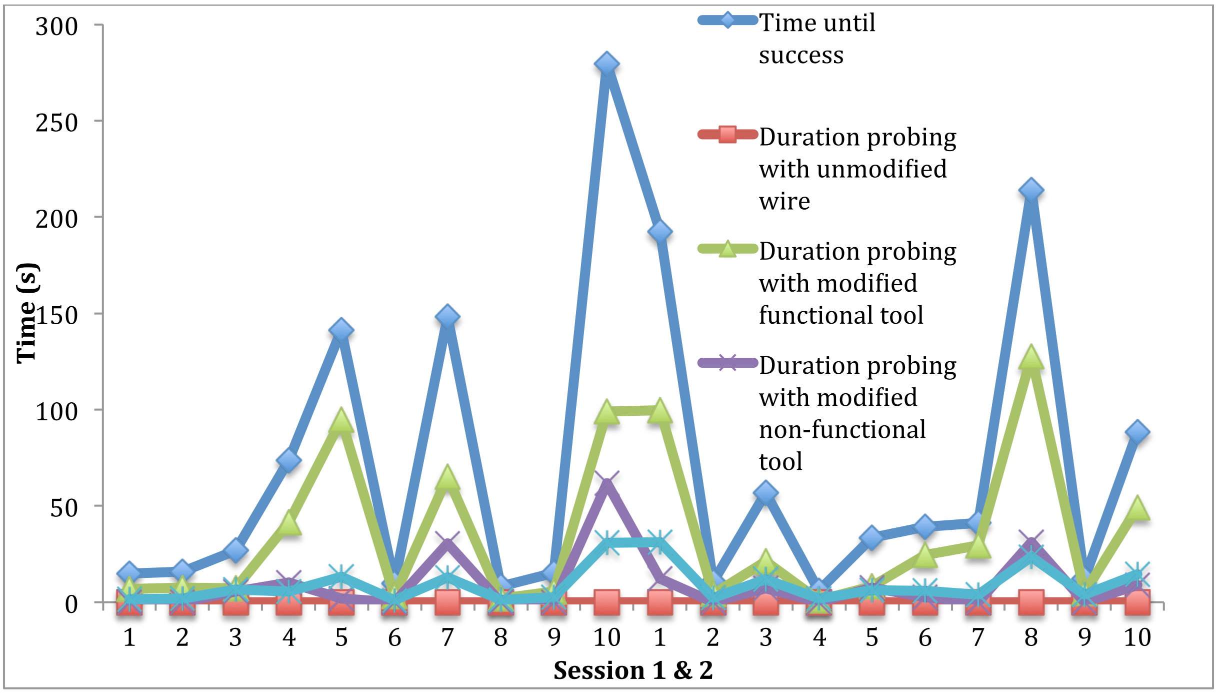

Fig. S3 Results of Padana´s successful trials in the hook-bending task with fixed basket.

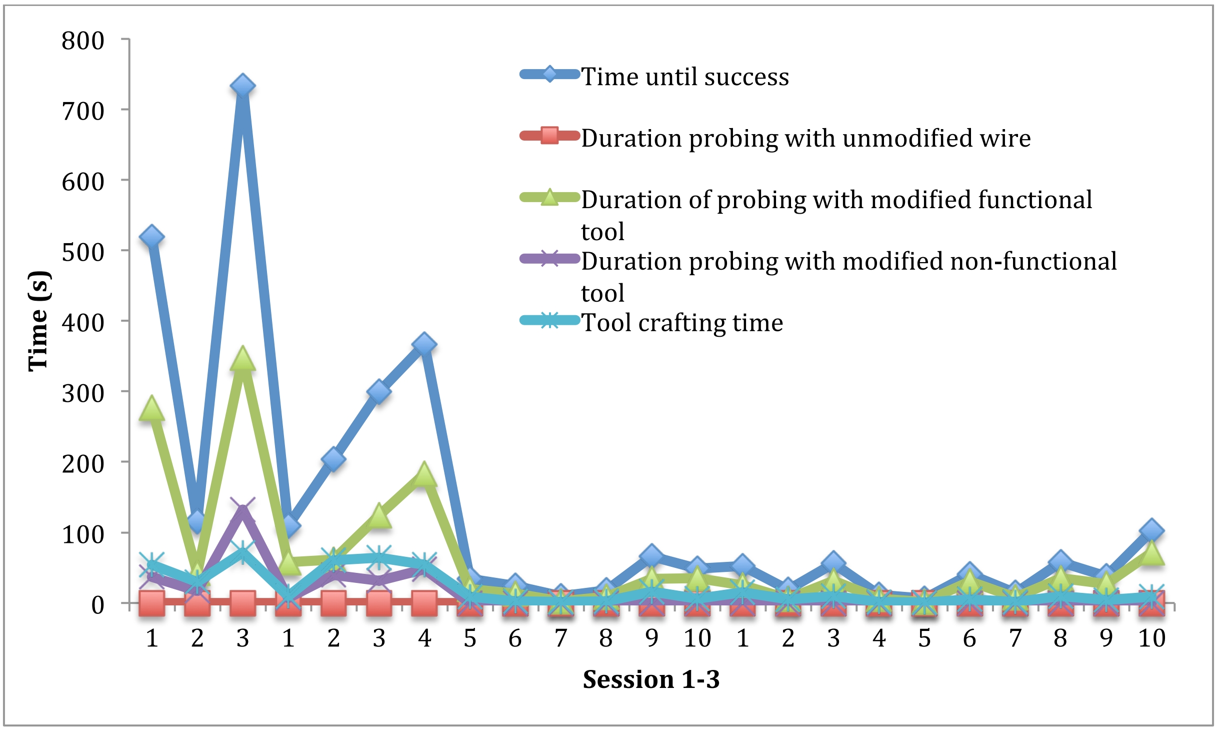

Fig. S4 Results of Pini´s successful trials in the hook-bending task with fixed basket.

d) Descriptive results of successful trials in the unbending task

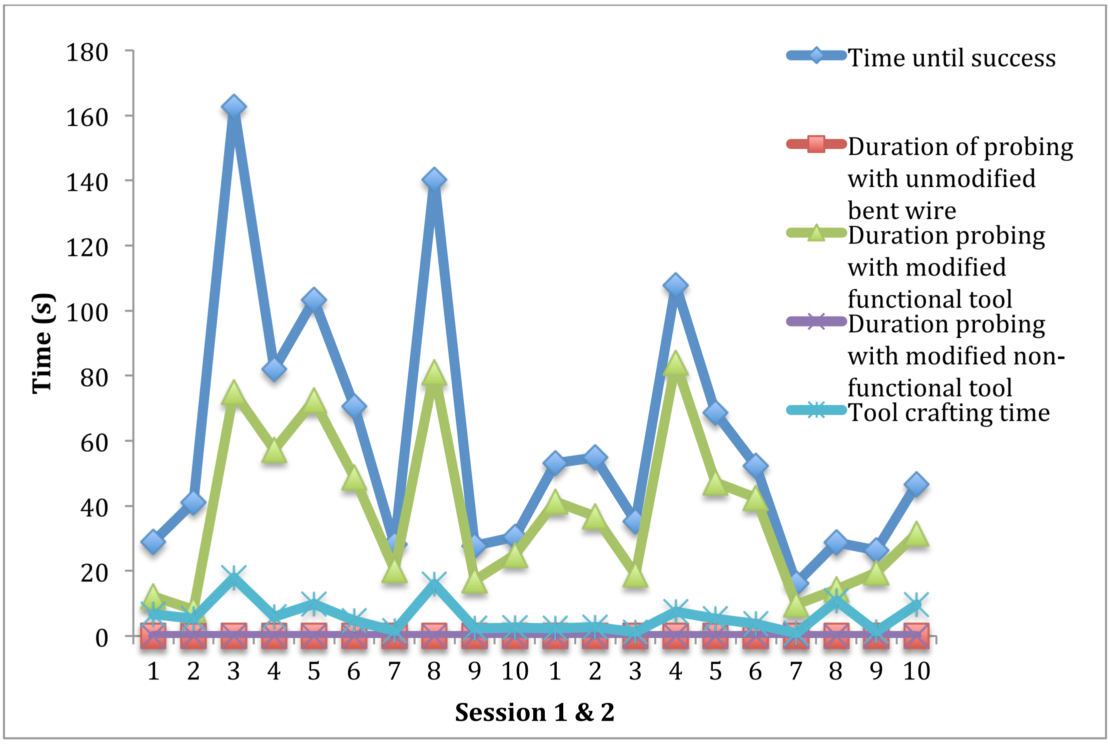

Fig. S5 Results of Padana´s successful trials in the unbending task.

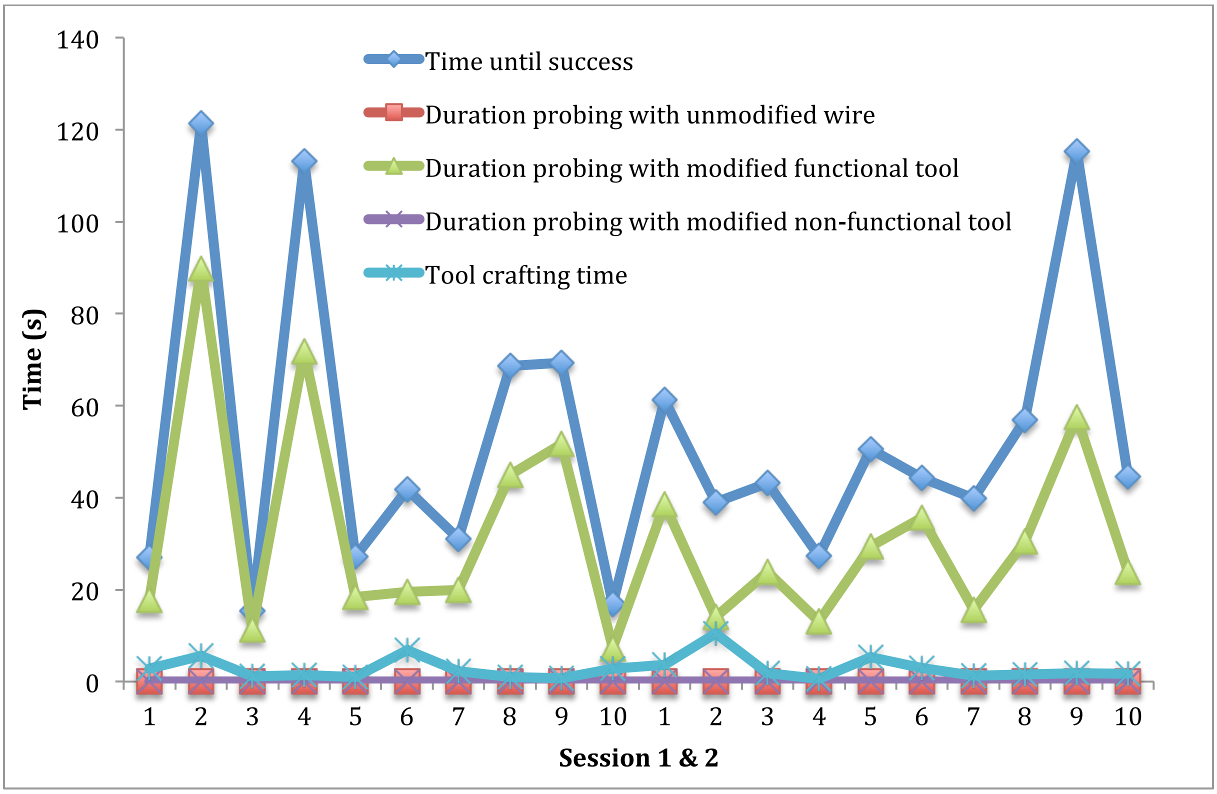

Fig. S6 Results of Pini´s successful trials in the unbending task.

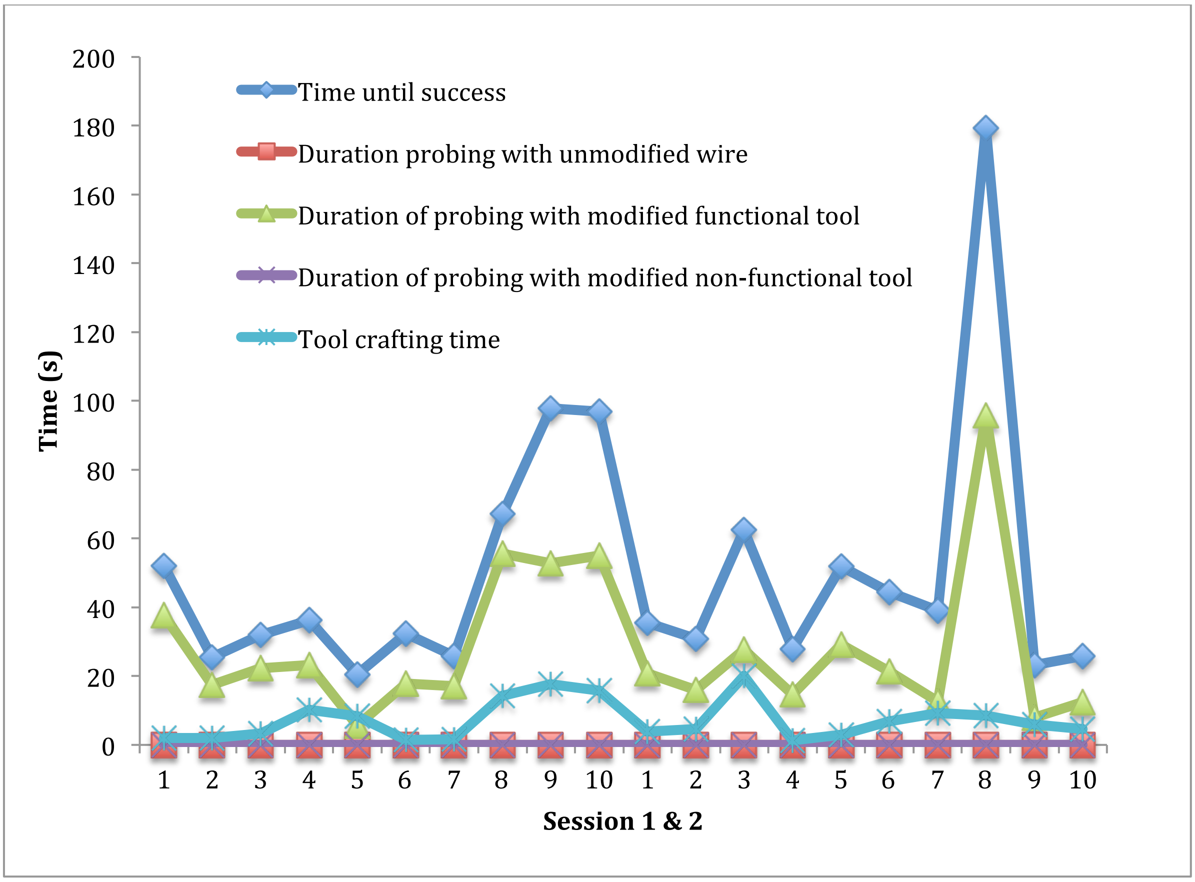

Fig. S7 Results of Dokana´s successful trials in the unbending task.

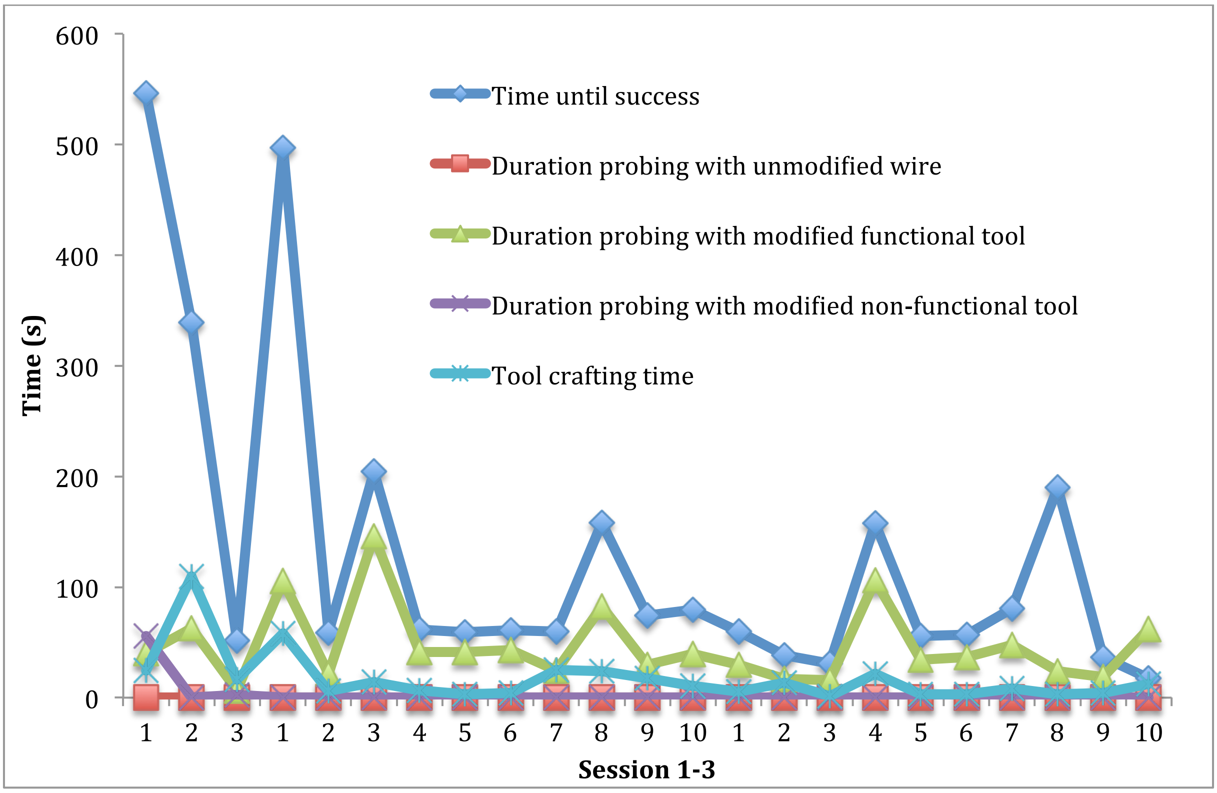

Fig. S8 Results of Raja´s successful trials in the unbending task.

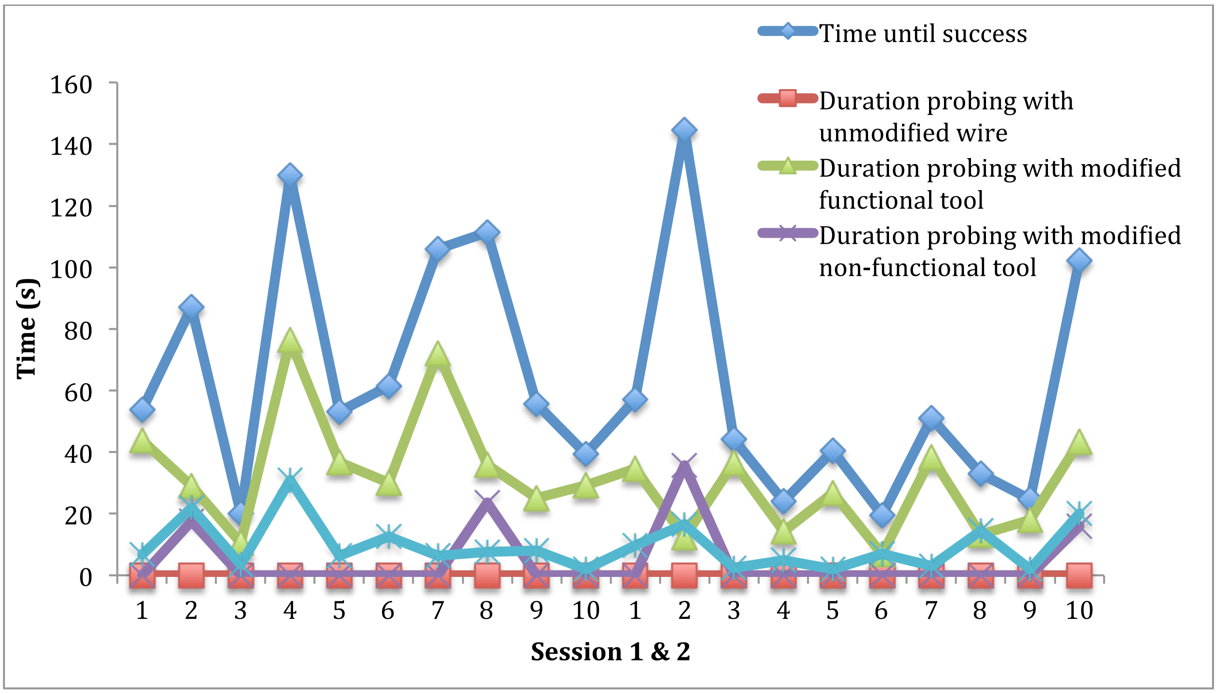

Fig. S9 Results of Bimbo´s successful trials in the unbending task.

e) Results of Raja´s and Tanah´s successful trials in the hook bending task

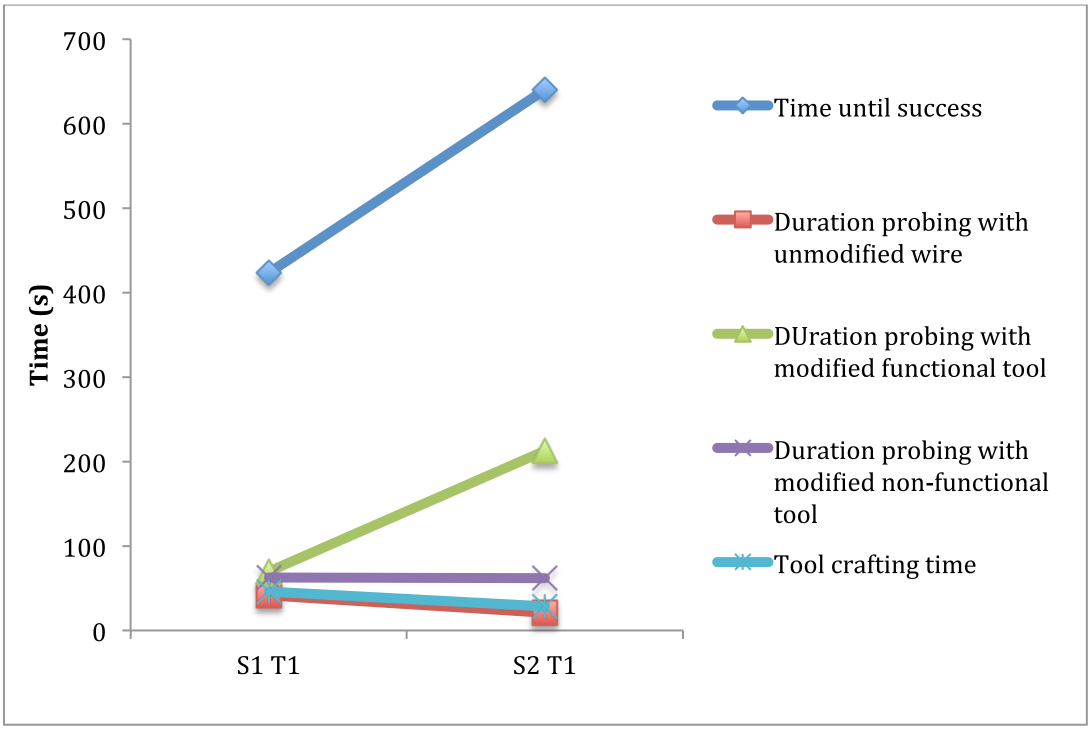

Fig. S10 Results of Raja´s successful trials in the vertical condition, tested after receiving PI&PP pre-experience.

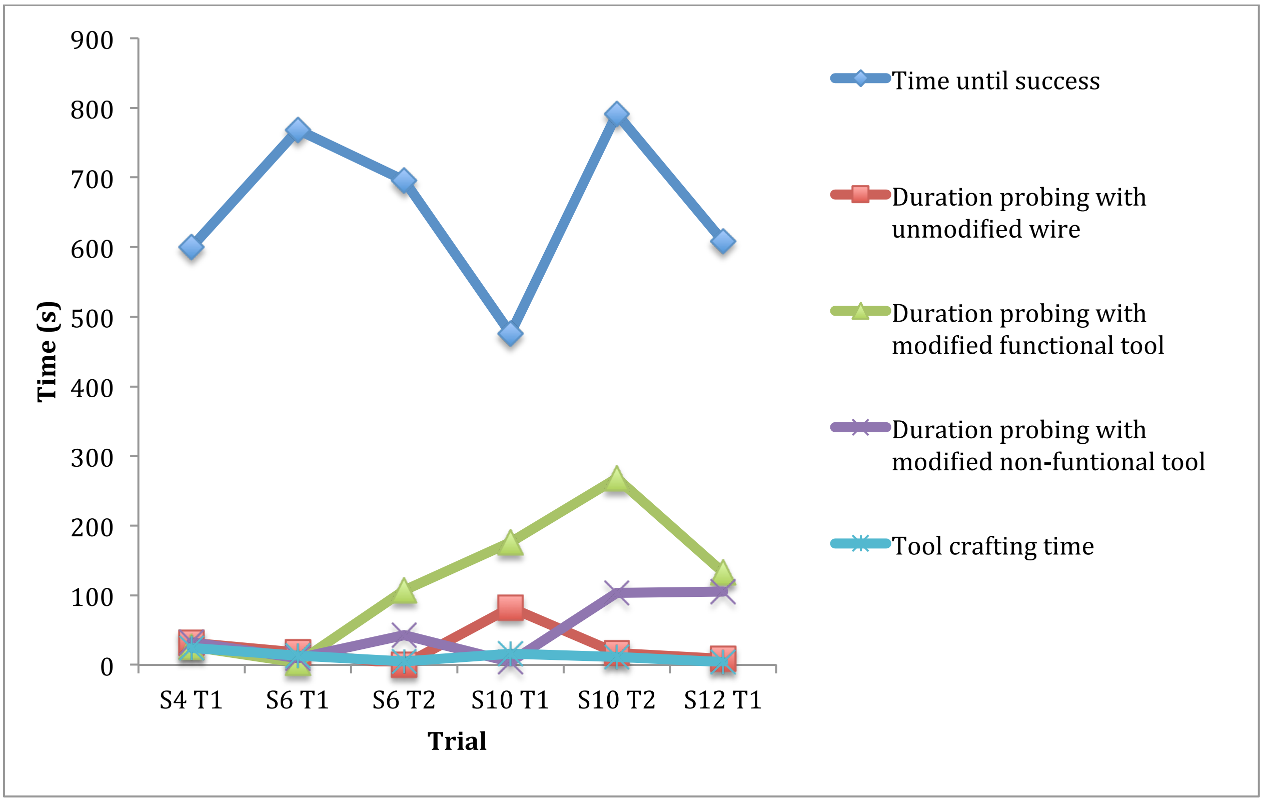

Fig. S11 Results of Tanah´s successful trials in the hook bending task .

f) Subject´s performance in other tool use tasks

We extracted the data from seven studies on various forms of tool use (water tool, avoiding traps, selecting rigid tools, see table S3). We then transformed their scores into ranks (shown in the table) so that we could get an idea of where Padana and Pini stand in relation to other orangutans at the Wolfgang Köhler Primate Research Centre in Leipzig. Their adjusted ranks are 1 and 3 in terms of tool proficiency, which fits well with the hook data. This is based on at least 10 conditions. We did not consider orangutans with less than ten conditions in the final ranking, (for instance Toba and Kila which only have one and three conditions, respectively).

A. Mendes, N., Hanus, D. & Call, J. (2007). Raising the level: Orangutans use water as a tool. Biology Letters, 3, 453-455.
B. Martin-Ordas, G., Call, J. & Colmenares, F. (2008). Tubes, tables and traps: great apes solve two functionally-equivalent trap tasks but show no evidence of transfer across tasks. *Animal Cognition, 11,* 423-430.
C. Martin-Ordas, G. & Call, J. (2009). Assessing generalization within and between trap tasks in the great apes. *International Journal of Comparative Psychology, 22*, 43-60.

D. Girndt, A., Meier, T. & Call, J. (2008). Task constraints mask great apes' ability to solve the trap-table task. *Journal of Experimental Psychology: Animal Behavior Processes, 34,* 54-62.
E. Marín Manrique, H., Gross, A.N. & Call, J. (2010). Great apes select tools based on their rigidity. Journal *of Experimental Psychology: Animal Behavior Processes, 36,* 409-422.
F. Marín Manrique, H. & Call, J. (2011). Spontaneous use of tools as straws in great apes. *Animal Cognition, 14*, 213-226.

Table S3 Ranks for each of the seven studies. The lower the rank the better the performance.

Study	A	B		C		D				E			F	rank	Adjusted rank	# cond
Condition		plat	tube	func	fake	Exp1	Exp1	Exp2	Exp2	str	tab	can				
Padana	3	2	3.5			4	4.5	2.5	3	3	2.5	3	2	3	3	11
Pini	3	3	1.5	2	1	2	1	2.5	1	3	2.5	3	4	2.3	1	13
Raja																
Bimbo		4.5	5			5	3	5	5	3	2.5	1	5.5	4.0	5	10
Dokana	3	1	1.5	1	2	3	2	1	4	3	5	3	2	2.4	2	13
Dunja	3	4.5	3.5			1	4.5	4	2	3	2.5	5	5.5	3.5	4	11
Toba	3													3	--	1
Kila		6	6										2	4.7	--	3
